# Vitamin A Update: Forms, Sources, Kinetics, Detection, Function, Deficiency, Therapeutic Use and Toxicity

**DOI:** 10.3390/nu13051703

**Published:** 2021-05-18

**Authors:** Alejandro Carazo, Kateřina Macáková, Kateřina Matoušová, Lenka Kujovská Krčmová, Michele Protti, Přemysl Mladěnka

**Affiliations:** 1 Department of Pharmacology and Toxicology, Faculty of Pharmacy, Charles University, Akademika Heyrovského 1203, 500 05 Hradec Králové, Czech Republic; mladenkap@faf.cuni.cz; 2 Department of Pharmacognosy, Faculty of Pharmacy, Charles University, Akademika Heyrovského 1203, 500 05 Hradec Králové, Czech Republic; macakovak@faf.cuni.cz; 3 Department of Clinical Biochemistry and Diagnostics, University Hospital Hradec Králové, Sokolská 581, 500 05 Hradec Králové, Czech Republic; katerina.matousova@fnhk.cz (K.M.); KRCML1AA@faf.cuni.cz (L.K.K.); 4 Department of Analytical Chemistry, Faculty of Pharmacy, Charles University, Akademika Heyrovského 1203, 500 05 Hradec Králové, Czech Republic; 5 The Department of Pharmacy and Biotechnology (FaBiT), Alma Mater Studiorum–University of Bologna, Via Belmeloro 6, 40126 Bologna, Italy; michele.protti2@unibo.it

**Keywords:** retinol, retinoic acid, retinoid receptor, vision, gene regulation, toxicity, hypovitaminosis, cancer

## Abstract

Vitamin A is a group of vital micronutrients widely present in the human diet. Animal-based products are a rich source of the retinyl ester form of the vitamin, while vegetables and fruits contain carotenoids, most of which are provitamin A. Vitamin A plays a key role in the correct functioning of multiple physiological functions. The human organism can metabolize natural forms of vitamin A and provitamin A into biologically active forms (retinol, retinal, retinoic acid), which interact with multiple molecular targets, including nuclear receptors, opsin in the retina and, according to the latest research, also some enzymes. In this review, we aim to provide a complex view on the present knowledge about vitamin A ranging from its sources through its physiological functions to consequences of its deficiency and metabolic fate up to possible pharmacological administration and potential toxicity. Current analytical methods used for its detection in real samples are included as well.

## 1. Introduction and Forms of Vitamin A

**Figure 1:**
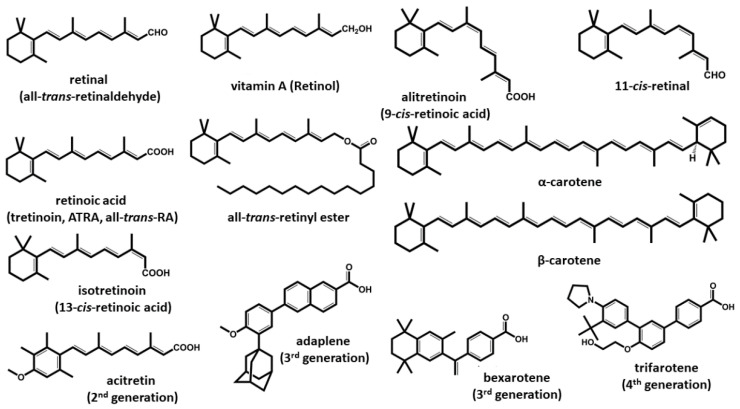
The structure of vitamin A and retinoids. The retinoids represented belong to the four described generations. First-generation compounds are found in the diet, except for some natural metabolites formed in the body. Members of the 2nd, 3rd and 4th generations are synthetic derivates based on the original retinoic structure and are used in treating different diseases. All retinoids possess a common structure and similar physicochemical properties, although their effects on the human body can vary greatly.

Vitamin A is a fat-soluble life-essential group of compounds both of animal and vegetal origin characterized by an unsaturated isoprenoid chain structure. All vitamin A forms share a similar structure and the same physiological functions in an organism. These compounds can also be classified as retinoids, including compounds with a common structure of four isoprenoid units being of either a natural or synthetic origin (Figure 1). Some synthetic derivatives do not resemble the natural isoprenoids from vitamin A class at first sight. However, the basic vitamin A string is hidden in their structures, and they are similar to other retinoids in their interaction with retinoid receptors. All these compounds are liposoluble and, unlike water-soluble vitamins, are easily accumulated in the body, especially in the liver and adipose tissue.This represents, on one hand, an advantage since temporal deprivation of vitamin A intake is not associated with clinical symptoms, but on the other hand, accumulation with subsequent toxicity can appear.

The vitamin can be provided in the diet either via products of an animal origin in the form of vitamin A (retinol and its close derivatives) or as provitamin A (carotenoids) from vegetables [1]. Although the term vitamin A is mostly associated with retinol, and retinol is, in fact, the predominant form of retinoids in the human body, the main biologically active molecules are the oxidized derivates 11-*cis*-retinal and all-*trans*-retinoid acid (ATRA) [1,2].

Carotenoids are yellow- to orange-colored organic pigments found in several fruit and vegetables. In addition to their relationship to vitamin A, they are known for their antioxidant activities. Some of the most well-known carotenoids are β-carotene, α-carotene, lutein, lycopene and cryptoxanthin. Carotenoids are tetraterpenoids in contrast to animal-origin diterpenoid retinoids but can be eventually metabolized to retinol. However, not all carotenoids can be converted into vitamin A in the human body. Only those molecules, which contain at least one unsubstituted β-ionone ring, have the nature of provitamin A.

Given the pleiotropic functions of retinoids, synthetic derivates have been developed, and therefore, retinoids can be classified into four generations. First-generation retinoids are forms found in nature: retinol, retinal, ATRA (tretinoin), 9-*cis*-retinoic acid (alitretinoin) and 13-*cis*-retinoic acid (isotretinoin). The second-generation retinoids were developed from the first generation, and the members of this group are etretinate and acitretin [3]. The third-generation of retinoids include adapalene, tazarotene and bexarotene [4,5,6,7]. Trifarotene is the only member of the fourth retinoid generation so far and has been approved only in the US [8]. Many of the compounds of all three classes are used clinically to some extent, with multiple indications, which are discussed in the corresponding sections of this review.

## 2. Sources of Vitamin A

The human body is not able to produce vitamin A, and therefore, it is necessary to obtain it from the diet either as preformed vitamin A or in the form of provitamin A carotenoids. There are more than 50 provitamin A carotenoids, but only β-carotene, α-carotene, and β-cryptoxanthin are present in significant amounts in the human diet [9]. These carotenoids have been identified in all groups of photosynthetic organisms, bacteria, fungi, and many animals [10]. β-Carotene is the most abundant in the diet. It is mostly ingested through red and orange vegetables and partially through the same colored fruits and green vegetables (Table 1). In Europe, carrots, spinach, and tomato products are the main contributors to β-carotene intake, while β-cryptoxanthin is most commonly taken from various citruses and citrus juices [11,12]. β-Cryptoxanthin, as well as other carotenoids, occurs in plants both as free and esterified with fatty acids (lauric, myristic, palmitic), and these esters contribute to total vitamin A content due to their comparable bioavailability [13]. Rich sources of cryptoxanthin or its esters include, in addition to the already-mentioned citruses (satsuma mandarins, tangerines, clementines, mineolas and oranges), persimmons, chili peppers and red peppers, papaya, sea buckthorn, loquat, mango and apricots [12,14,15,16]. Other sources of provitamin A carotenoids also include various medicinal plants and herbs, cereals, and specific vegetable oils (Table 1).

The content of carotenoids in individual plant species varies greatly due to many factors. One of the main indicators may be the color of the flesh of a particular cultivar of fruit or vegetable. A clear connection has been shown, for example, in sweet potatoes and winter squash [17,18]. There is also a strong correlation between the degree of ripeness and the carotenoid content of fruit. The highest content of carotenoids is found in fully ripe fruit [19,20,21]. Of course, other factors can also have an effect, such as the method and location of cultivation [19,22]. All of the above apply to fresh fruit and vegetables, but most species are processed or modified in various ways for better digestibility or storability. Many kinds of fruit, vegetables and herbs are dried before storage, and the method of drying used has been shown to have a large influence on the final content of carotenoids. Up to 90% losses can occur in sun-dried material, while up to 70% carotenoids can be retained when dried in a tray dryer at high temperatures (up to 80 °C) [23,24,25]. As for conventional heat treatment methods, bleaching and cooking are comparable, maintaining an average of 75% carotenoids, whereas frying is less gentle on carotenoid content but still retains more than one-half of the content [21,25,26]. Jams are a common product of various kinds of fruit processing. The loss of β-carotene in these preparations is comparable to conventional cooking, even when using a microwave oven [27].

The bioavailability of β-carotene from fruit and vegetables is significantly lower than purified β-carotene (by one order of magnitude) and differs significantly between species, which may result from differences in the intracellular location of carotenoids. Heat treatment has the potential to increase bioavailability [28,29]. The oldest method used to obtain pure β-carotene is extracting it from plant material. The main disadvantages of this method are its high price and the seasonality of the resources. Another option is synthetic production. However, carotenoids prepared in this way were questioned concerning their safety, and, therefore, a number of methods have been developed for the biotechnological production of “natural” β-carotene. Many microscopic organisms, including yeasts, molds, algae, cyanobacteria and bacteria, can produce β-carotene and other carotenoids. The main commercial source is the alga *Dunaliella salina* (Dunal) Theodoresco. Another species used on an industrial scale is the mold *Blakeslea trispora* Thaxter [29,30,31]. β-cryptoxanthin is produced by green algae and bacteria. However, it is not used for industrial production [32,33,34].

An important source of the human intake of vitamin A is the preformed version of the vitamin, which is found in foods of an animal origin. Milk and dairy products, as well as meat and its products, are the largest contributors, followed by eggs, egg products and fish [11]. The amount of retinol found in the milk of individual livestock species does not differ greatly, although feed, seasonal variation and breeds do have some impact. In general, its concentration ranges from 20 to 80 µg per 100 mL of whole milk. Each breed produces milk with a different amount of fat related to the amount of vitamin A in its milk. The higher the fat content in the milk, the higher the retinol content [49,50,51,52,53,54]. The amount of retinol in dairy products depends on the amount of milk fat in the product [55]. One of the richest sources of vitamin A is the livers of various livestock and poultry, which contain tens of milligrams of retinol and its esters per 100 g. Differences between animal species are significantly more pronounced than in the case of milk. The values found in individual studies vary considerably, but the highest vitamin A content in the liver is usually reported in pigs [56,57,58,59,60]. The amount of vitamin A is significantly dependent on the amount of β-carotene contained in the feed or food supplements with preformed vitamin or β-carotene given to the animals. Large differences can be observed, for example, in cows fed a diet based mainly on grains or that are grass-fed [59,60]. The content of vitamin A is significantly lower in other animal organs, and it reaches only units up to tens of micrograms per 100 g in meat [58,61]. The same is true for both freshwater and sea fish. Their livers also contain units up to tens of milligrams of vitamin A per 100 g (some tropical fish even up to hundreds), while their muscles contain only tens of micrograms per 100 g. As with other animals, the highest vitamin A levels are found in the livers of the top predators, i.e., carnivorous fish. One of the most important sources of vitamin A is the liver oil of various marine species, including sharks. According to the European Pharmacopoeia, cod liver oil contains 18–75 mg of vitamin A per 100 g of oil. The content of vitamin A in various shark liver oils reaches similar values. A significant amount of the vitamin is also found in fish eggs, where the amount is comparable to chicken egg yolks [62,63,64,65]. Chicken egg yolks can contain up to almost one milligram of vitamin A per 100 mg. Some sources have stated that duck eggs are poorer in vitamin A than chicken eggs, but others have reported the opposite. However, the yolk of quail eggs can contain up to twice the content of that of chickens [36,55,66]. The discrepancies in individual studies might be due to many factors. For example, there are significant differences between individual breeds of hens and molt programs [67]. Efforts to increase the amount of preformed vitamin A in eggs through a high-carotenoid diet (biofortified corn, red palm oil or citrus peels) have not increased retinol content in eggs, despite significantly increased carotenoid levels. However, the content of provitamin A carotenoids in eggs is insignificant compared to the preformed vitamin A content [68,69,70].

## 3. Vitamin A Pharmacokinetics

### 3.1. Absorption, Distribution, Metabolism and Elimination

#### 3.1.1. Absorption

Vitamin A is mainly obtained orally from the diet. However, when used as a drug, additional routes of administration, including both intramuscular and topical ways, are possible. Although retinoids have a common structure consisting of a hydrophobic region, a linker, and a polar region, understandably, the pharmacokinetic profile depends on each way of administration, on the physic–chemical characteristics of each vitamin form and the pharmaceutical form used.

**Figure 2:**
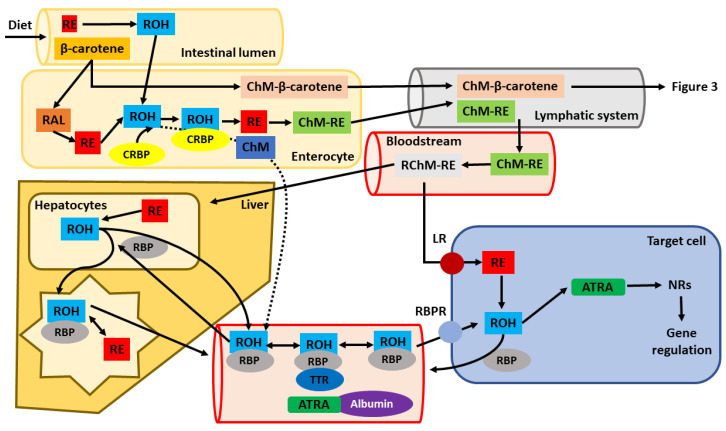
Cellular pathway, uptake and transport of orally given vitamin A. After metabolization in the intestinal lumen, penetration into enterocytes and its association to chylomicrons (ChM), retinyl esters (RE) and β-carotene are secreted into the lymphatic system. Later, they reach the blood (systemic circulation) and are subsequently delivered to the liver, which functions as the main retinoid storage organ in the body or target tissues/cells. The dashed line represents the portion of retinol, which is not metabolized in the intestinal cells into retinyl esters and is secreted directly into the bloodstream, where it can bind to retinol-binding protein (RBP). From the liver, retinoids can be directly secreted into the blood in association with RBP or bind later to other transport proteins (e.g., albumin) found in the blood. Transport to target tissues is enabled via the RBP-receptor (RBPR). Once they enter the target cells, retinyl esters or retinol (ROH) are further oxidized into all-*trans*-retinoic acid (ATRA), which is responsible for the genetic functions of vitamin A in the body (other abbreviations: RAL—retinal; RChM-RE—remnant chylomicrons-retinyl esters; TTR—transthyretin; LR—lipoprotein receptor; NRs—nuclear receptors; CRBP—cellular retinol-binding protein).

Since the oral route is the most frequent (Figure 2), this pathway is described in detail. Absorption differs significantly between animal vitamin A (retinol and its derivatives) and carotenoids [1,71]. The retinyl esters found in animal-origin foods are almost completely absorbed, while carotenoid absorption is significantly lower.

As most animal-based vitamin A takes the form of retinyl esters, these esters reach the intestine. Before entering the enterocytes, they are metabolized into retinol by a triglyceride lipase or phospholipase B in the lumen of the gastrointestinal tract (Figure 2). Retinol uptake can take place via active transport or by passive diffusion [72]. Its absorption is increased if consumed with fatty meals since micelle formation supports the absorption of fat-soluble compounds, such as animal vitamin A in the small intestine [71]. In addition to fat, some micronutrients, such as zinc, are also needed for the absorption of the vitamin [73]. Once in the enterocyte, retinol binds to a specific protein called cellular retinol-binding protein (CRBP), responsible for the intracellular transport of retinol [74,75]. To date, two isoforms of this protein have been characterized, CRBPI and CRBPII. The main difference between them is their different expression within the body. Whereas isoform CRBPI is widely expressed, isoform CRBPII is mostly limited to intestinal cells, emphasizing its key role in the absorption process [76].

Carotenoid intestinal absorption was originally believed to happen through passive diffusion. However, further research has demonstrated the involvement of the scavenger receptor class B1 (SCARB1) transporter and the cluster of differentiation 36 (CD36 or SCARB3) proteins, although it is still believed that a portion of carotenoids can be absorbed by passive diffusion [77].

**Figure 3:**
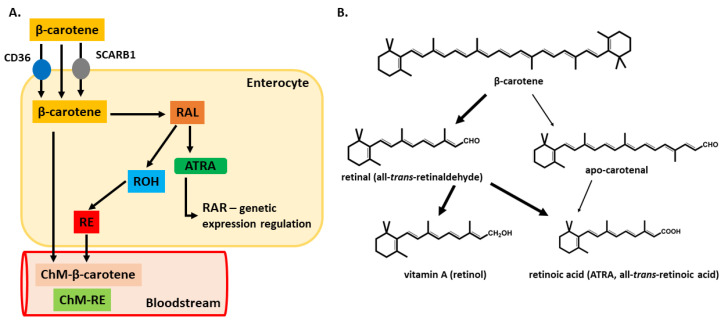
β-carotene metabolizing pathways. Absorption in the intestinal lumen can happen through passive diffusion, or it can be mediated by the membrane proteins SCARB1 and CD36. Once in the enterocyte cytoplasm, there are two possible metabolization routes. Part **A** shows the most common metabolization pathway y, leading finally to the secretion of retinyl esters (RE) or β-carotene into the bloodstream associated with chylomicrons. On the right (part **B**), both metabolic pathways are illustrated, the common route and the alternative cleavage yielding apo-carotenal molecule. Both molecules have the same metabolic end product (all-trans-RA, ATRA). The thick arrows indicate the most common pathways, while the thin arrows indicate less common metabolizing routes. Abbreviations: SCARB1—scavenger receptor class B1; RAL—retinal; ROH–retinol; CD36—cluster of differentiation 36; ChM-β—carotene–chylomicron-β-carotene; ChM-RE—chylomicron-RE).

Carotenoids in intestinal cells can be metabolized into biologically active forms of vitamin A or pass through in their unaltered form (Figure 3A). Early studies suggested that approximately one-half of carotenoids were absorbed in their unaltered form, while the rest was metabolized into retinol [78]. However, only about one-third of administered carotenoids, mostly β-carotene, can be metabolized in the intestinal epithelia. The conversion rate depends on different factors: their amount, retinol levels in the body and fat content of the diet [79]. In addition, an alternative cleavage procedure has been described in which β-carotene can be metabolized into β-apo-carotenal (Figure 3) [80,81]. Retinal formed from β-carotene is further oxidized into ATRA or reduced to retinol. This process is not specific to the enterocyte. It can also happen in the liver and other organs. β-carotene metabolism is carefully regulated through feedback mechanisms, clearly emphasizing its importance as a human source of vitamin A [82,83,84]. When carotene intake is high, there is only a small conversion rate, and most of the carotene is stored in the adipose tissue and other fat reserves.

The subsequent fate of absorbed or formed retinol is logically the same regardless of its origin [85]. It is metabolized into retinyl esters, mostly palmitate, in the enterocytes and secreted within chylomicrons into the lymphatic system [1,86,87,88]. Once in this complex, they are not accumulated in the liver. Some of the esters are metabolized back into retinol, and the remaining esters are further transported in association with very-low-density lipoproteins (VLDL) and low-density lipoproteins (LDL) [89]. As mentioned, they can be de-esterified into retinol and further metabolized into active forms of vitamin A in different cells.

Apart from retinol and retinyl esters, other retinoids can also be present in lower concentrations in the blood thanks to being directly absorbed through the portal circulation, such as ATRA or isotretinoin [90,91]. ATRA has low bioavailability after p.o. administration. It has a very high affinity to plasma proteins, and hence it is transported in a complex formed with albumin after reaching the blood (Figure 2). Isotretinoin, a first-generation retinoid, is only used orally and has a bioavailability of around 20%. Isotretinoin is also extensively bound to albumin in plasma. Tissue concentration is usually lower than that in plasma. Etretinate and acitretin, which are second-generation retinoids, are also used in oral form in therapy. They have a bioavailability of approximately 50%.

#### 3.1.2. Distribution and Cytoplasmic Fate of Vitamin A

Chylomicrons containing retinyl esters formed in the enterocytes eventually reach the bloodstream, from where they can get to the target tissues, mainly the liver, which is the main storage organ of vitamin A in the body [92]. However, before retinyl ester-loaded chylomicrons reach the liver, they undergo several enzymatic reactions, which lead to the formation of chylomicron remnants, which are later taken up by the liver [93]. Once in the liver, a portion of retinyl esters are hydrolyzed into retinol, and in this form, they associate with RBP, a highly abundant protein synthesized in hepatocytes, but, which is also present in several other tissues, such as adipose tissue, the lungs, kidneys, eyes and testes [1,94,95]. The formation of this complex leads to it being secreted from the liver into the bloodstream and subsequent distribution throughout the body [76]. Once in the bloodstream, the retinol-RBP complex associates with another liver protein, transthyretin (TTR). TTR is better known for its thyroid hormone transport function but apparently also plays a role in the kinetics of vitamin A. In fact, its name is derived from “transports thyroxine and retinol”. TTR can bind to RBP before being secreted into the circulatory system, forming a stable complex needed for the correct delivery of retinol to target cells and avoiding the degradation of RBP in the kidneys [96,97,98,99,100]. The retinol–RBP complex is taken up by target cells through plasmalemmal transporters, which play a key role in recognizing retinol blood transporters and in the cellular uptake of retinol (Figure 2) [101,102,103]. Peripheral tissues have a substantial need for vitamin A. In fact, almost one-third of the retinoids end up in different organs (the kidneys, bone marrow, skeletal muscles, adipose tissue, etc.) [1,104]. The concentration of retinol-RBP complex is quite stable unless severe vitamin A deficiency is present. Under these conditions, the amount of RBP is decreased due to the decreased levels of vitamin A in the body. Not all retinol in the hepatocytes is secreted with RBP. Some retinol is transported to the stellate cells of the liver, where it is metabolized into retinyl esters and forms the main storage of vitamin A in the body [93,105]. In general, vitamin A is stored in the form of retinyl esters [106]. In addition, adipose tissue and other organs can store retinoids. Examples include the testes, adrenal glands, lungs, kidneys and interstitial cells. This storage ability is important for organs with a high vitamin A requirement, especially retinal epithelium. In cases of a vitamin A-deficient diet, physiological levels can be maintained for several months thanks to these stored reserves. When needed, these storage organs release retinoids into the blood. Before entering the circulatory system, esters are hydrolyzed, and up to 95% of the retinol is associated with transporting proteins. Interestingly, acitretin is quite water-soluble and, therefore, is not markedly stored in adipose tissue [107,108].

Circulating retinoids are usually bound to blood proteins, especially albumin and, as already mentioned, RBP. These complexes can be taken up by target cells, e.g., through the mediation of lipoprotein receptors or being stimulated by the retinoic acid 6 receptor (STRA6) [109].

Retinol located in target cells is mainly used for forming ATRA. In this pathway, retinol is first metabolized into retinal by alcohol dehydrogenases, and it later binds to CRBP. In the second step, retinal dehydrogenases further oxidize retinal into ATRA, which then binds to the cellular retinoic acid-binding protein (CRABP). ATRA, the acidic form of vitamin A, is the final product of vitamin A oxidation and cannot be further reduced back into retinal or retinol and cannot be stored.

#### 3.1.3. Elimination

The elimination of retinoids happens through the kidneys or via the liver into bile [110]. The evacuation of stored retinoids formed in the body is slower than water-soluble vitamins due to their being stored in different organs. After intake interruption, months can go by before noticing any vitamin A deficiency.

### 3.2. Other Factors Influencing Vitamin A Pharmacokinetics

Drugs and (patho)physiological states can directly influence the absorption of the vitamin. Drugs such as estrogens and oral contraceptives have been reported to elevate plasma concentrations of RBP, thereby elevating retinoid blood levels [111]. Alcohol consumption is an important factor mediating the inhibition of vitamin A metabolism. In alcoholic liver disease, retinol concentrations are significantly decreased [112].

### 3.3. Pharmacokinetics of Vitamin A during Pregnancy

During gestation, retinol concentration in the plasma decreases during the first trimester and slowly increases back, again reaching normal values before delivery [113]. Circulating retinoids in the mother (retinol and retinyl esters) provide vitamin A to the fetus and must be transported through the placental barrier after dissociation from transporting proteins [114,115,116]. The transfer of RBP from the mother to the fetus occurs only in the first trimester. Later, the fetus can synthesize its own RBP. Although the placenta has been believed to protect the fetus against high-vitamin A intake from pregnant women being over-supplemented with vitamin A, several studies have reported that high vitamin A intake by the mother can lead to teratogenic effects in the developing fetus [117,118,119].

## 4. Vitamin A Functions

Vitamin A has pleiotropic functions in the body, thanks to its several biologically active forms. Although retinol, which is also responsible for some processes, is the most abundant form in the body, ATRA is the major active form of vitamin A [1,120,121,122,123,124]. To a lesser extent, other metabolites of this vitamin, 9-*cis*-retinoic acid and 13-*cis*-retinol, are also biologically active. Each form of the vitamin shows specificity for different tissues and processes in which they are involved. However, they share similar common properties [125]. Retinol acts as a cofactor in several enzymatic processes, 11-*cis*-retinal is involved in vision, and ATRA exerts different functions by binding to nuclear receptors with the subsequent regulation of genetic expression.

**Figure 4:**
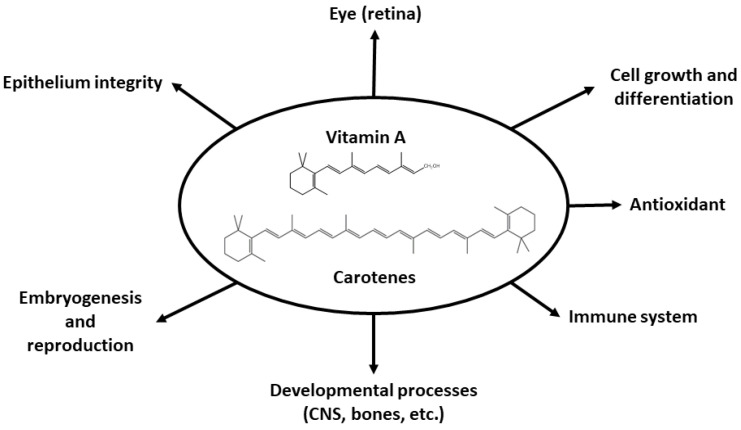
A schematic representation of the physiological roles in which vitamin A is involved.

A summary of the wide range of physiological processes in which retinoids are involved is provided in Figure 4. These processes include vision in darkness, corneal and conjunctiva development, cellular growth and differentiation, immune system functioning, bone and fetus development and central nervous system (CNS) formation. Carotenoids also act as antioxidants [126,127,128,129,130,131,132].

Interestingly, retinoids have been reported to also be involved in several pathological situations: cardiovascular diseases, diabetes mellitus, obesity, osteoporosis, skin diseases and cancer [103,133,134,135,136,137,138,139]. The role of vitamin A in each process is described in the following sections.

### 4.1. Vision

**Figure 5:**
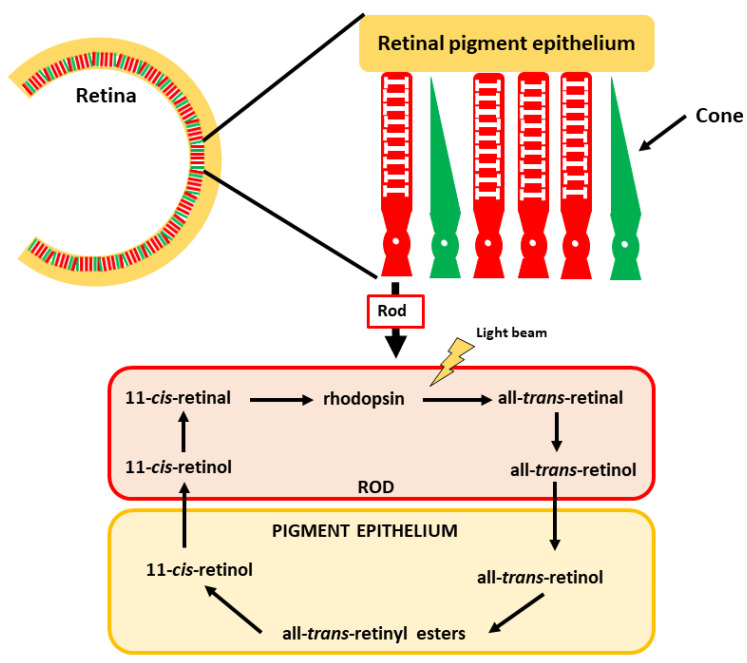
Vision and the role of 11-*cis*-retinal in the process. The retina comprises cones and rods, which mediate color and low light vision, respectively. The vitamin A derivative 11-*cis*-retinal is found in the rods, forming rhodopsin.

In the eye, the retina is the structure responsible for visual perception, including its transmission to the brain. This perception is mediated by specific structures in the retina: rods and cones. Rods are sensitive to low light and hence are crucial for vision in dark situations (e.g., night vision), whereas cones are responsible for high-intensity light (color vision). The active vitamin A derivate is 11-*cis*-retinal in this case. It is associated with the protein opsin, a G-coupled protein receptor in the retina. The complex is known as rhodopsin, which is the crucial pigment for light perception [140,141]. Upon light stimuli, 11-*cis*-retinal is transformed into all-*trans*-retinal and initiates a chain of reactions whose ultimate consequence is transmitting optic perceptions via the optic nerve to the brain (Figure 5). After this reaction, some all-*trans*-retinal can be transformed back into 11-*cis*-retinal, enabling recycling of this key molecule. The remaining all-*trans*-retinal can be transformed into retinol, which can be stored in the epithelial cells to be later reused or converted into ATRA [142,143].

A deficiency in retinol leads to low light vision impairment due to deficient rhodopsin formation. This situation causes night blindness, which is also called nyctalopia [144]. Low light vision can be recovered after the normalization of plasma retinol levels. However, it takes several weeks until the normal function is completely restored.

### 4.2. Interaction with Nuclear Receptors

Vitamin A is an important factor in gene regulation [145,146]. This effect is exerted through interaction with nuclear receptors (NRs). NRs are ligand-activated transcription factors that, upon ligand-binding, can modulate the target gene expression through direct interaction with DNA. Although 48 families of NRs have been described to date in humans, all members show a common structure consisting of a DNA-binding domain (DBD), a ligand-binding domain (LBD) and a hinge region that connects both structures. LBD is usually a highly specific structure that recognizes the ligands for each type of receptor. These receptors can be activated both by endogenous molecules and/or xenobiotics [147].

Retinoids are known to interact with several families of nuclear receptors. In 1987, the first receptor with a high affinity for a retinoid was identified. It was named the retinoic acid receptor α (RARα) due to its ability to bind retinoic acid (ATRA) with a high-affinity [148,149]. This breakthrough discovery explained the mechanisms of some biological functions associated with retinoids. Since then, additional receptors have been described that interact with retinoids. These receptors are commonly called retinoid receptors. The main retinoid receptor families are RAR and retinoid X receptor (RXR), but a third retinoid-interacting NR family has been described: RAR-related orphan receptor (ROR).

**Figure 6:**
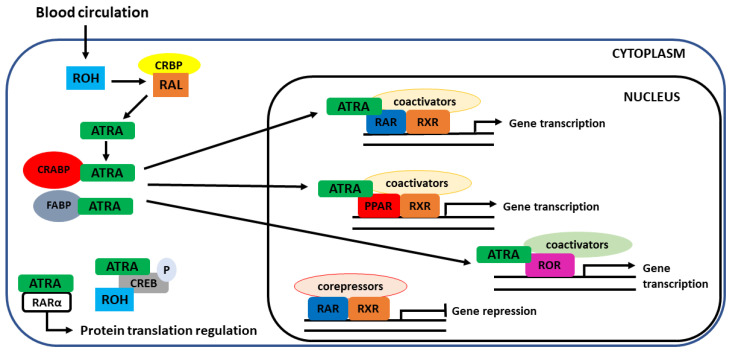
Cellular uptake by target cells and intracellular receptors for vitamin A. Once in the cytoplasm, retinol undergoes several oxidation steps, which end up forming ATRA, which can follow different fates inside the cell. ATRA can mediate both genomic and non-genomic functions. The non-genomic functions are less known and include regulation of phosphorylation of target proteins (CREB) and cytoplasmic translation regulation. Genomic functions are more common and include the binding of ATRA to nuclear receptors (RAR, PPAR, RXR, ROR), which have a direct influence on gene regulation. In the absence of a ligand, gene transcription is repressed. For this to happen, ATRA must be transported to the nucleus, which is mediated by cellular retinoic acid-binding proteins (CRABP) or fatty-acid-binding protein (FABP). CREB—cAMP response element-binding protein.

After the discovery of the first member of the RAR family, RARα (NR1B1), two more RAR isoforms were successfully isolated and reported to interact with vitamin A derivates: RARβ (NR1B2) and RARγ (NR1B3) [148,149,150,151]. RARs strongly bind to ATRA, as well as to 9-*cis*-retinoic acid (Figure 6). The expression of these receptors is tissue-specific. RARα is widely distributed throughout the body, whereas isoform RARβ is predominantly in the brain, liver and kidneys. RARγ is highly expressed in the epidermis [152]. RARs play different roles, both genomic and non-genomic. In general, they are involved in cell signaling. The non-genomic processes are mediated through phosphorylation processes [153] and the ability of RARα to regulate protein translation [154].

The second family of receptors with a high affinity for retinoids is the RXR. This receptor represents the key for the functioning of RAR and many other nuclear receptors since its presence is necessary for forming heterodimers and, subsequently, the transcriptional machinery. RXRs were described for the first time several years later than RAR [155]. Similar to RAR, RXR also presents three isoforms with different distribution within the body (α-NR2B1, β-NR2B2 and γ-NR2B3) [155,156,157]. Whereas RXRα is mainly localized in the liver, lungs and intestines, RXRβ is ubiquitously distributed throughout the body and RXRγ is predominantly found in muscles and the brain [152]. The three isoforms are important heterodimerization partners for fellow nuclear receptors, mainly from the NR subfamily 1. The main difference between the RAR and RXR families lies in the LBD structure, which determines ligand specificity. ATRA is a high-affinity ligand for RAR but can also activate RXR. However, the retinoid with the highest affinity for RXR is 9-*cis*-retinoic acid [152,158].

Although these two closely related receptor families share the same or similar ligands and have similar signaling pathways, they regulate a different set of genes.

At the molecular level, in the absence of ligands, RAR is found in the cellular nucleus forming a complex with co-repressors. In this form, RAR is inactive. Upon binding to a ligand, the complex releases co-repressors by mediating a conformational change and co-activators are recruited [159,160,161]. RAR needs to heterodimerize with RXR to form the transcription machinery complex, promoting gene transcription (Figure 6). The heterodimer-co-activator-ligand complex recognizes specific DNA sequences for binding to the promoter region of target genes called retinoic acid-responsive elements (RARE). RXR can heterodimerize with more NRs, such as pregnane X receptor (PXR), constitutive androstane receptor (CAR) and vitamin D receptor (VDR), among others [147,162].

The last NRs to which retinoids can bind are RORs. As in the RAR and RXR families, the ROR family also presents three isoforms: α, β and γ, and again, in this case, their expression is tissue-specific. Therefore, RORα is mainly found in the liver and the brain, β in the retina and the brain, and γ in the liver and the testes, among other tissues [163]. The main difference from the other described retinoid receptors is that they can regulate gene expression in monomers through binding to the ROR responsive elements (RORE) and hence do not need dimerization with a fellow retinoid receptor RXR [164]. RORs bind to oxysterols with high specificity. However, constitutive activation of the receptor, in the absence of a ligand, has also been described [165]. ATRA is the retinoid with the highest affinity for RORβ [166,167].

In addition to the receptors described thus far, another family of receptors has been described that interact with retinoids, peroxisome proliferative activated receptors (PPARs) (Figure 6). This has been documented mainly for ATRA [168]. As in the case of the other retinoid receptors, this receptor family also presents three isoforms α (NR1C1), β/δ (NR1C2) and γ (NR1C3) [169,170]. To exert their regulatory activity, these receptors also heterodimerize after ligand binding with RXR to form a transcriptional complex. Highly specific ligands for these receptors are fatty acids, and they are involved in energy homeostasis, fatty acid metabolism and inflammation [171,172,173,174,175]. However, only isoform β/δ presents high-affinity for ATRA [176,177]. This isoform is abundantly expressed in the skin, brain and adipose tissue. The discovery of the interaction between ATRA with PPARβ/δ explains the proliferative effect of RA in keratinocytes and its involvement in insulin sensitivity and energy homeostasis [168,178].

Interestingly, ATRA can regulate its levels in target organs through catabolic processes, especially through interaction with the enzymes CYP26A1, CYP26B1 and CYP26C1 [179,180,181,182]. The role of ATRA in NR interaction is the most frequently studied mechanism after the visual role of vitamin A. The genetic effects triggered by interactions between ATRA and its receptors are directly involved in multiple physiological functions: cellular differentiation, tissue development, tissue regeneration, cellular apoptosis, etc. [120,121,122,124]. In addition, ATRA has additional functions in gene regulation since the evidence shows it has non-coding RNA regulatory functions [183].

#### 4.2.1. Vitamin A and Cancer

The role of retinoids in cancer has been the focus of many studies. However, no conclusive relationship has been clearly established. Retinoids are known to promote cell growth and tissue development through their interaction with NRs. Therefore, some studies have suggested that retinoids can be considered as cancer-promoting compounds. Although antineoplastic activity is related to the RAR transrepression of activating proteins, ATRA has been suggested to have antineoplastic effects even though it is a receptor activator [184,185,186,187]. In vitro studies have demonstrated the effects of retinoids on apoptotic genes [188,189] and the potential protective effect of vitamin A against some types of cancer [190,191]. This information follows a study that reported vitamin A deficiency as a factor in cancer development [125]. In addition, retinol has been suggested to also be involved in regulating cell growth [187]. ATRA, used in treating acute promyelocytic leukemia, promotes cell differentiation by activating transcription factors. However, it also inhibits a set of proteins involved in cell development and activates cellular apoptosis [127,192]. In addition to its differentiating effect, it has also been reported to inhibit the proliferation of lymphoma and lung, liver and solid ovarian tumors [7,185,193]. Importantly, in addition to tretinoin and alitretinoin, other generation retinoids are already being used clinically as antiproliferative agents [4,194].

The role of carotenoids in cancers is even more controversial, especially in the case of β-carotene. Although being a well-known antioxidant with potentially positive effects in cardiovascular diseases and type II diabetes mellitus, several studies have reported contradictory effects of carotenoids on cancer incidence. Lower incidence of epithelial and lung cancer has been observed in various studies in individuals with a high intake of carotenoids from diets rich in fruit and vegetables [195,196]. However, some studies have reported that in a cohort of smokers, a higher incidence of lung cancer and mortality rate was observed after the administration of β-carotene when compared to the control group [197,198,199]. It is important to point out that these effects were observed with β-carotene supplementation, not with lower carotenoid intake ingested through the diet [200]. Contrarily, several clinical and preclinical studies have reported carotenoids to prevent generating reactive oxygen species (ROS), to induce apoptosis in tumor cells and to prevent cancer induction [201,202,203].

#### 4.2.2. Vitamin A, Immunity and Inflammation

The adaptative immune system is another process in which vitamin A plays a key role. The vitamin acts as a cofactor in the proliferation and differentiation of regulatory T cells and several immune functions through indirect processes [204,205,206,207]. In the presence of vitamin A, IL-2 levels are increased, which stimulates the differentiation of T cells into regulatory T cells, which are important mediators for the prevention of autoimmune responses [129,208]. Regulatory T cells can modulate forming the transforming growth factor-β (TGF-β), a key complex in immune and inflammatory reactions, whose expression can be modified by RA [209]. Without this intercommunication, the sole presence of TGF-β would facilitate an autoimmune response. Despite the roles already reported for ATRA in the immune system, contradictory roles for retinoids have been reported regarding its role in inhibiting or enhancing inflammatory reactions [210,211]. Importantly, lymphocytes present retinoid receptors at their surface, which recognize ATRA and retinol. The retinoid role is apparently linked to more than just retinoid receptors since retinol, but not ATRA, acts as a cofactor for B lymphocyte growth and T lymphocyte activation. The effects are mediated especially by its metabolite 14-hydroxy-4,14-retro-retinol (14-HRR) [212,213]. In addition, 14-HRR has been reported to have similar growth-promoting effects in fibroblasts and promyelocytes. In addition to T cell differentiation, vitamin A is also important for regulating hematopoietic stem cell dormancy and other inflammatory mediators. In states of deficiency, the population of hematopoietic stem cells decreases since they cannot remain dormant, leading to imbalances in the immune system [186,214]. The immune system’s response to infections, such as measles and parasitic infections, is compromised in vitamin A deficiency, and the severity and length of these conditions are prolonged if the levels of vitamin A are not quickly restored [186].

#### 4.2.3. Other Functions Associated with Gene Transcription

Vitamin A is also involved in mucin synthesis by the goblet cells in the intestine [215]. An early study performed in chickens determined that goblet replacement rate and, therefore, mucin formation decreases in vitamin A deficiency [216]. This process is especially observed in GIT epithelium. A well-known function of vitamin A is to maintain the functional and structural integrity of the epithelium in different tissues. Retinoids participate in maintaining normal epithelial homeostasis by promoting the differentiation of keratinocytes into mature epidermal cells. With adequate vitamin A levels, basal epithelial cells in mucus-secreting or keratinizing tissues are stimulated and produce mucus [215,217]. Deviations in retinoid homeostasis have a direct effect on skin integrity [218]. Under conditions of excessive retinoid concentrations, a thick layer of mucin is produced, inhibiting keratinization, leading to deleterious effects. On the other hand, in vitamin A deficiency, mucous secretion is suppressed and causes stratification and keratinization of the epithelium, leading to irritation and subsequent infection. The skin, sweat glands, eyes, trachea, bronchi, salivary glands and genitourinary tract are affected by this deficiency [218]. These effects of vitamin A exerted at the epidermal level are mediated by the interaction of ATRA with NRs, mainly RAR and RXR, which are expressed in keratinocytes, hair follicles, and dermal fibroblasts [219].

Embryogenesis is another process in which the presence of retinoid is crucial for correct growth and development [122,220,221,222]. During pregnancy, the mother needs to provide vitamin A to the fetus. Vitamin A deficiency in this period leads to embryonal malformations, known collectively as vitamin A deficiency syndrome, which is manifested by cardiovascular and nervous system deficiencies and less developed tissues, among other defects.

After birth, newborns are usually born with low vitamin A levels even though their mothers commonly have values within the recommended ranges [223]. These levels are corrected through breastfeeding since breast milk is a rich source of vitamin A [224]. Vitamin A requirements in breastfeeding women are logically increased. The main retinoid found in breast milk is ATRA since it is slightly more water-soluble than the other retinoids [2].

In wounded tissues, retinoids promote epidermal turnover and normal tissue restoration. ATRA mediates these functions by promoting the synthesis of collagen and fibronectin and the proliferation of keratinocytes, supporting the rapid restoration of a normal epidermis [225]. In retinoid deficiency, normal epithelium recovery is impaired, and the process needs a longer time to occur.

Through NR activation, retinoids are involved in lipid metabolism and insulin sensitivity. The activation of RAR and PPAR regulates genes that are directly involved in glucose transport, fatty acid oxidation, lipolysis and adipocyte differentiation [178,226,227]. Elevated RBP levels in the blood have been linked with insulin resistance [228]. Contrarily, retinal has been reported to improve metabolic syndrome by preventing adipose tissue generation, playing a direct role in obesity [229]. Decreased levels of vitamin A have been recently linked with mortality in the senior population [230]. In obese individuals, adipose tissue is widely spread throughout the body, compromising the correct functioning of multiple physiological processes since this tissue is a rich source of cytokines, hormones, and growth factors. A recent study has linked a higher plasma level of retinoids with lower cardiovascular disease risk in diabetes mellitus type II patients [231]. Additionally, it is believed that carotenoid intake helps in preventing obesity. However, further research on the topic is needed [232,233,234]. Additional processes in which vitamin A involvement has been reported are lipid metabolism, insulin response and energy homeostasis [235,236,237].

Meiotic entry is another process in which retinoids are involved through their interaction with NR, especially RAR. This process, which encompasses the pass between mitosis and meiosis, is essential in gonadal differentiation in fetal development and in spermatogenesis in adult males. ATRA has been reported to be the triggering factor in the meiotic entry process in animal models and humans [238,239,240]. This is a consequence of the function of ATRA in cell differentiation processes. However, this process is highly specific among species [241]. Alterations in the levels of factors involved in this process or in the onset of timing lead to consequences, which may be quite serious for human health, such as sex development disorders, infertility, or even forming cancer [242].

### 4.3. Other Functions of Retinoids

This section summarizes other physiological or pharmacological activities, which do not seem to be related to their interaction with retinoid receptors.

Since the levels of vitamin A circulating throughout the body are higher than the amount of vitamin needed for vision and the already reported genetic functions, it is understandable to suggest that retinoids can be involved in additional biological processes. In the second half of the last century, a decreased activity of several enzymes was observed in vitamin A deficiency, indicating that retinoids can act as cofactors in some enzymatic reactions [215,243,244]. These non-genomic activities of retinoids explain many of their activities, such as the effects observed at the dermatological level [245]. The non-genomic effects can be mediated via protein phosphorylation, which continues with genomic activation [153,246].

Vitamin A participates in reduction–oxidation homeostasis [247,248]. The first retinoid form to be described to act in this way was retinol, which was reported to bind to different proteins from the serine/threonine kinase family, specifically rapidly accelerated fibrosarcoma (Raf) and protein kinase C (PKC), and function as a redox reagent [249,250]. In addition to retinol, ATRA is known to regulate the activity of these enzymes, which are involved in proliferation and differentiation [251,252]. Carotenoids, as reported above, are well-known antioxidants [253,254]. However, research has indicated that in excess, carotenoids may have pro-oxidant effects as well [255,256].

Age-related macular degeneration is a frequent cause of blindness in the senior population. This condition is associated with oxidative stress. Therefore, compounds with antioxidant properties, such as carotenoids, have been tested in treating this illness. Recent studies have reported that intake of carotenoids lutein and zeaxanthin, but not β-carotene, showed a lower risk of developing this illness [257,258]. Since β-carotene is not involved, this effect is likely not vitamin A-based. Additionally, carotenoids have also been reported to be potentially able to improve diabetic retinopathy [259,260].

Another process in which vitamin A, or more precisely, ATRA, is involved is non-genomic rapid synaptic transmission (166). ATRA has also been reported to inhibit Ca-ATPase activation mediated by thyroxine (T4) and 3,3’,5-L-tri-iodothyronine (T3) enucleation of erythrocytes [261]. Retinoids have also been reported to be active at the CNS level. ATRA has been suggested to be involved in memory development and learning processes [131,262]. This role has been confirmed by the deficiencies observed in CNS structural abnormalities and impaired development in situations of ATRA absence [120]. Interestingly, a recent study has linked the potential positive use of retinoids in Alzheimer’s disease, probably through cell differentiation regulation [247].

ATRA also has been shown to have additional extranuclear functions, such as kinase activation (e.g., MAPK). An alternative mechanism for the activity of retinoids has been suggested to take place through interactions with proteins by covalent bonds. Studies have reported that although a scarce number of proteins can act in this way, some of them are highly relevant for physiological processes in which important enzymes, such as cAMP-kinase and ribonucleotide reductases, to name a few, are involved [263,264].

Retinoids also play a role in bone homeostasis [265,266]. Elevated levels of retinoids have been described to have undesirable effects in bones in experimental animals by promoting their fragility and thinning [267,268]. However, decreased levels of vitamin A have deleterious effects on bone metabolism as well [269]. On the other hand, carotenoids have been reported to contribute to correct bone formation via their antioxidant properties. However, such effects are not related to the physiological function of vitamin A [265]. In addition, crosstalk between vitamins has been described regarding bone metabolism, which is understandable, mainly among the fat-soluble vitamins A, D, E and K. Several examples confirm this finding, e.g., (1) Vitamin A has been reported to prevent vitamin D and E absorption in in vitro models [270], (2). Although it is well-known that decreased vitamin D promotes bone fragility, this effect has been reported to be worsened in individuals also presenting a high intake of vitamin A [271,272,273] and similarly, high vitamin A levels decrease calcium absorption by mediating the vitamin D-calcium response [274,275], (3). A high vitamin A intake has been proposed to decrease vitamin D toxicity and toxic effects on bone metabolism [276], (4). In addition, vitamin D deficiency and a high blood concentration of vitamin A have been linked with bone fragility [272], (5). A potential synergetic effect between both vitamins causing apoptosis in cancer cells and preventing lung cancer development, has also been suggested [277,278]. Therefore, vitamin A levels in the body may have consequences regarding the metabolism of other vitamins or in the effects produced by them.

## 5. Analytical Approaches for Measuring Vitamin A Levels

Currently, several analytical instrumental methodologies with the needed sensitivity, specificity, and/or resolution to quantify endogenous retinoids and related compounds in tissues and biological fluids are available (Table 2 and Table S1). The analysis of retinoids should address crucial requirements that must be considered to ensure reliable qualitative–quantitative results. In fact, to have sufficient sensitivity and specificity to detect the analytes in physiological conditions and carefully validated protocols, making a quantitative determination of retinoids in biological samples requires separative techniques capable of resolving endogenous isomers.

The retinoid analytic methods described in the literature include (ultra) high-performance liquid chromatography ((U)HPLC) coupled with spectrophotometric (UV-Vis/DAD), spectrofluorimetric (FLD), electrochemical (ECD) detection, mass spectrometry (MS and MS/MS), and also supercritical fluid chromatography (SFC) and immunoassay-based ones, featuring different sensitivities, effectiveness towards different biological matrices, benefits, and limitations. A comparison of these methodologies is provided in Table 2 [279,280,281,282,283,284,285,286,287,288,289,290,291,292,293,294,295,296,297,298,299,300,301,302,303,304,305,306,307,308,309,310,311,312,313,314,315,316,317,318,319,320,321,322,323,324].

### 5.1. Detection Means

HPLC methodologies coupled with UV-vis/PDA detection are usually feasible and cost-effective but provide medium-low sensitivity and do not allow for mass identification. However, recent advances in chromatographic column technologies have at least partially allowed for lower detection limits. UV after HPLC separation of retinoids offers analysis specificity to some extent because few compounds absorb at the characteristic wavelengths of retinoids. Additionally, UV detection of retinoids shows a certain degree of structure-dependent absorbance maxima, potentially providing additional information. The benefits of UV also include simplicity and cost-effectiveness, particularly when compared to MS-based detection methods. Whereas single wavelength and DAD are effective for in vitro retinoid assays and quantitation of abundant endogenous retinoids (retinal, retinol, retinoid esters) in vivo, they usually lack the needed sensitivity for making an endogenous bioanalysis of retinoid acid. In fact, concentrations of endogenous ATRA in tissues are up to several orders of magnitude below the limit of detection and/or limit of quantification for both DAD and single-wavelength detection [279,280,281,282,283,284,285,286,287,288,289,290,291,292,293,294,295,320,321,322,323,324].

Fluorescence spectroscopy-based detectors offer greater specificity than spectrophotometric ones (because of selecting both excitation and emission wavelengths) for analyzing retinol and its analogs in biological samples. Retinol and retinyl esters are intensely fluorescent but retinal, and retinoic acid, together with most synthetic retinoids, do not show any fluorescence. On the other hand, as regards carotenoids, only a few show any appreciable fluorescence. For this reason, analytical platforms coupled with fluorescence detection are not heavily exploited for performing routine retinoid and carotenoid analyses [296,297].

HPLC methods coupled with ECD possess enhanced sensitivity but lack the definite mass identification of MS analytes. They can be affected by interference from matrix compounds and other analytes, and their sensitivity is strongly influenced by solvents, electrode type and flow characteristics [308,309,310].

GC–MS allows for good sensitivity but requires additional derivatization steps for making retinoid acid analyses, often representing labor-intensive procedures and a potential source of errors. Single-quadrupole, LC–MS-based assays offer mass identification of analytes but do not have the enhanced sensitivity and specificity of selected reaction monitoring (SRM) or multiple-reaction monitoring (MRM) modes provided by triple quadrupole MS/MS detection. Triple-quadrupole LC–MS/MS facilitates the most effective qualitative–quantitative analysis of retinoic acid with sufficient sensitivity and specificity, yet no requirement for derivatization or definite mass identification. A low abundance of endogenous ATRA requires sensitive detection. MS/MS is currently the most sensitive method used for making bioanalyses of retinoic acid and is readily coupled with LC separations capable of resolving isomers of retinoic acid. MS/MS offers appropriate sensitivity for the detection through background reductions of 100–1000-fold over MS. MS/MS also provides enhanced selectivity by requiring analytes to meet both parent ion and product ion *m/z* conditions for detection. The sensitivity and background reduction advantage of MS/MS allows for performing analyses of small amounts of tissue and biological samples with more reliable qualitative–quantitative data. Information obtained from MS/MS fragmentation can also identify unknown molecules [298,299,300,301,302,303].

### 5.2. Chromatographic Considerations

Because retinoic acid isomers are isobaric and have overlapping UV spectral profiles, single-quadrupole mass and/or single wavelength UV detection cannot distinguish between geometric isomers that may co-elute. Several instrumental methodologies allow for the separation of endogenous isomers of retinoic acid. Methods for resolving such isomers include both normal-phase and reversed-phase separation approaches, and reversed-phase has become predominant due to its more feasible coupling with MS/MS. Methods based on the reversed-phase separation of endogenous isomers of retinoic acid mainly involve using C18 stationary phases or bonded stationary phases with embedded polar groups. For example, amides embedded in the bonded phase increase the selectivity of the chromatographic column towards polar analytes and have been reported to perform better than classic C18 for separating retinoic acid isomers.

SFC instrumental methodologies use CO_2_ in its supercritical state as the main eluting solvent. The elution strength is then modulated by the addition of small amounts of modifiers, which in most cases is alcohol, or to a lesser extent, buffers or acids/bases to extend the range of sample polarity that can be analyzed. Compared to both reversed- and normal-phase solvents, CO_2_ is non-flammable, cheap, miscible with common organic solvents, and is less polluting. This is the main reason why SFC can be considered a “green” chromatography technique. In terms of system performance, supercritical CO_2_ has low viscosity and high diffusivity, which leads to reduced equilibration and analysis times. Finally, CO_2_ is transparent to radiation above 190 nm, allowing for UV and fluorescence detection. One of the main limitations of SFC is sample solubility as highly polar compounds, such as ionic ones, are difficult to analyze [304,305,306,307].

### 5.3. Other Methods

Besides the instrumental analytical methodologies mentioned above, additional methods based on immunoassays have also been developed for assessing retinoids and carotenoids. Enzyme-linked immunosorbent assays (ELISA) are the most widely used immunoassays due to their high sample throughput. These methods can drastically reduce the number of analyses required to detect vitamins in different samples. Such methods are generally justified when there is a need to carry out routine quality control of samples with rather simple compositions. The advantages of these methods include their simplicity of use, compactness, and relatively low analysis costs. Immunoassays are ready for use and cost-effective for high-throughput analyses, making them particularly useful for routine uses. On the other hand, they may lack sufficient specificity, especially at the physiological levels, as they can show cross-reactivity among retinol analogs, while they are time and money consuming for small sample series [311,312,313,314,315,316,317,318,319].

### 5.4. Considerations on the Stability of Retinoic Acid

Retinoic acid is susceptible to degradation even when stored under temperature-controlled conditions. Thus, careful precautions must be taken to ensure high-quality sample handling protocols before making instrumental analyses since it can lead to artifactual changes in endogenous retinoid levels. More abundant endogenous retinoids, such as retinol and retinyl esters, are less susceptible to storage-induced degradation.

As general considerations when analyzing retinoic acid in tissues and biological fluids, samples should be kept frozen until homogenization (samples should not be stored as homogenates for prolonged periods). At the same time, rapid freezing procedures (e.g., by using liquid nitrogen) are advisable, shielding the samples from light exposure at all times during processing and storage as much as possible. Performing analyses within a few days from tissue harvesting or biological fluid collection is preferable, but it is possible to postpone it for up to 1–2 weeks without any significant degradation. Most samples stored for periods exceeding 1 month may show a measurable loss, especially for retinoic acid. Preliminary analyte matrix-dependent stability assays are advisable if storage before analysis occurs. Finally, freeze–thaw cycles should be avoided unless stability has been verified.

## 6. Vitamin A Deficiency

Intake recommendations on vitamin A vary according to the age of the individuals and are usually expressed as retinol activity equivalent (RAE), where one RAE equals 1 μg of retinol, 12 μg of β-carotene and 24 μg of α-carotene or β-cryptoxanthin [325]. For children and infants, the recommended vitamin A intake varies around 400–500 RAE. In adult males, the recommended daily intake is 900 RAE, while for females, pregnant and lactating women, the recommended levels vary between 700 and 1300 RAE, with the highest being for lactating women. As mentioned, adequate vitamin A levels are key factors for the correct development of the fetus and, later, for the correct alimentation of the newborn [326].

### 6.1. Symptoms

The most characteristic consequence of vitamin A deficiency is impaired vision. Early sight impairment is significant, especially under conditions of reduced light. In extreme cases, due to long-term vitamin A deficiency, the conjunctival and corneal epithelium losses their differentiation ability, leading to hyperkeratinization of the ocular epithelial tissue (xeropthalmia), and eventually, total blindness, which can be permanent. This circumstance is the most common cause of blindness in developing countries [142,144].

Epithelial modifications due to vitamin A deficiency directly affect several systems in the human body and cause weight loss. In the respiratory system, changes in the bronchorespiratory epithelium occur, and the tissue is more prone to infections. Skin can keratinize, and the epidermis dries out with the subsequent appearance of papular eruptions and keratinization of sweat glands. Epithelial modifications also occur in the urogenital system. At the reproductive system level, spermatogenesis is impaired, and testes degeneration can be observed. In the gastrointestinal tract, there is a reduction of the number of goblet cells in the intestines, epithelial alteration, and pancreatic ductal epithelium metaplasia. Nerve lesions have also been reported. Taste and smell functions are also partially mediated by vitamin A through the mediation of mucopolysaccharides synthesis, which are responsible for taste sensing [327], and the keratinization of this tissue leads to a loss of the sense [328,329,330]. In addition to a higher frequency of airway infections due to an impaired epithelium, general susceptibility to infection and inflammation are other symptoms of vitamin A deficiency. Malnourishment contributes to this phenomenon [331]. During inflammation, nutrient requirements are enhanced, and immunity is impaired [332]. In addition to low vitamin A content, decreased levels of blood transport proteins are also found in malnourished children, which further impairs vitamin A pharmacokinetics and subsequent functionality [333]. Vitamin A is also known to interfere with iron metabolism. Its deficiency has a direct impact on iron levels through metabolism and heme synthesis impairment. This is especially important in children and pregnant women [334]. Therefore, vitamin A deficiency can cause anemia. The role of vitamin A in carcinogenesis was described in Section 4.2.1 [125].

### 6.2. Causes and Epidemiology

Insufficient vitamin A levels are diagnosed when retinol plasma levels decrease below 0.52 µM, or liver concentrations are lower than 5–20 μg/g. They can be classified as primary or secondary deficiencies depending on the cause. An insufficient intake of vitamin A and carotenoids through the diet is the primary cause of the deficiency. It is typically present in developing countries. Symptoms usually follow a poor diet leading to malnutrition. Secondary deficiency is associated with lipid malabsorption and chronic diseases, including biliary tract insufficiency, cirrhosis, chronic diarrhea, sprue, Crohn’s disease, pancreatic insufficiency, etc.

Vitamin A deficiency is more prevalent worldwide than retinoid intoxication [335]. A balanced diet usually provides the required physiological levels of vitamin A. In cases in which recommended levels are not reached, vitamin A supplementation is required. This is of special importance in infants and children, where this intervention reduces the rate of child morbidity and mortality. To date, vitamin A supplementation in developing countries is considered one of the costliest interventions in improving child survival worldwide since it is calculated that approximately one-third of the world’s infant population suffers from hypovitaminosis A [335,336]. In addition to the child population, in developing countries, around 15% of all pregnant women suffer from this deficiency. The reason is very likely malnourishment [336,337]. About 500,000 children suffer annually from early blindness due to vitamin A deficiency. However, as mentioned above, the consequences of deficiency are much larger and include impaired immunity, frequent respiratory infections, enamel hypoplasia, skin illnesses, genitourinary and reproductive system defects and abnormalities in bone formation [333,338]. In light of the health consequences of vitamin A deficiency, it is understandable that a lack of vitamin A can be fatal. It is calculated that this deficiency is responsible for the death of more than 650,000 children/year in developing countries.

Subclinical deficiency of vitamin A can also appear and usually happens in diets poor in vegetables and meats. This is again highly relevant in less developed countries, but it can happen in developed areas too. Additionally, other factors can directly affect the metabolism and overall levels of vitamin A, for instance, alcohol intake. In pregnant women, alcohol intake is believed to be the cause of fetal alcohol syndrome, leading to teratogenicity, partly due to induced vitamin A deficiency [339]. However, an equilibrated diet usually provides sufficient levels of vitamin A and, generally, no supplementation is required, even during pregnancy [117].

Widespread vitamin A deficiency, especially in Sub-Saharan Africa and South Asia, has led to the development of many projects aimed at the biofortification of various widely consumed foods in these areas. Biofortification is achieved in various ways, such as breeding, agronomy, and genetic transformation. Foods targeted by the projects include rice, corn, sweet potatoes, cassava, and bananas. The most well-known is transgenic golden rice 2 containing up to 37 µg β-carotene per gram of rice, and it is already being grown in some countries [44,340].

## 7. Therapeutic Use of Vitamin A

Different retinoids (Figure 1) are used in various therapies. Apart from the well-known indications in hypovitaminosis and skin diseases, retinoids have been successfully used in treating cancer [6,7,185,187,193,341,342]. Whereas most retinoids are usually available in oral preparations, acitretin is indicated for topical use.

In addition to malnutrition, retinol supplementation is also indicated in treating measles, where the requirements of vitamin A are significantly increased. Although almost irrelevant in so-called developed countries, this infection is still one of the leading causes of death in developing countries. Therefore, the administration of supplements in these situations significantly increases the survival rate and improves vision disturbances [343,344,345]. Apart from measles, other infections involving GIT and respiratory system, cause a reduction in RBP synthesis, thus secondarily decreasing the circulating levels of vitamin A. In circumstances in which retinol storage or distribution may be affected (steatorrhea, biliary obstruction, cirrhosis, gastrectomy), long-term substitution therapy with retinol is indicated. Supplementation treatment may also be indicated in other circumstances in which retinol loss is observed, such as prolonged healing of injuries [346].

The FDA has approved ATRA to treat cystic acne and several cancers, such as some lymphoma and leukemia [6,187,341,347,348]. However, recently, the spectrum of indications has been expanded to include several solid tumors (liver, lung, melanoma, breast and prostate cancers) [342]. Other retinoids used in cancer treatment are bexarotene and alitretinoin. Importantly, developing resistance to ATRA treatment by tumors is common [193,349]. On the other hand, bexarotene can selectively bind to RXR and modulate gene expression and cell proliferation and has been successfully used in treating cutaneous T-cell lymphoma [7]. Alitretinoin has been used in Kaposi sarcoma, where other therapies have failed [350]. The major drawback of these synthetic derivates is their multiple adverse effects, which they share with other retinoids, and this problem can be more accentuated with bexarotene.

ATRA has multiple functions in the body and can be used therapeutically for different purposes depending on its method of administration. As a topical agent, it can be administered for acne and photoaging treatment [351,352]. In addition, the anti-inflammatory effect of ATRA can contribute to these effects [353].

Retinoids are frequently used in skin disorders: psoriasis, photodamage, seborrhea, acne, and ichthyosis. For these purposes, ATRA, isotretinoin, adapalene and acitretin are usually indicated [5,354,355,356,357]. However, several precautions should be considered by patients being treated with them: avoiding solar exposure, use of other topical treatments in the same area, delaying pregnancy, etc. In acne, isotretinoin is usually recommended due to its activity as a topical bactericide and its ability to reduce inflammation due to its inhibitory properties toward monocytes and neutrophils and its capacity for remodeling sebaceous glands.

Acitretin is an effective agent in psoriasis, even as a monotherapy. It is also used to treat discoid lupus due to its anti-inflammatory and antiproliferative effects [358]. However, as is the case with other retinoids, it can cause side effects [3].

Other expanded uses of retinoids are found in the cosmetics industry and as dietary supplements [359,360]. Several skin creams often contain retinyl palmitate, which can be absorbed and metabolized into retinol and further into ATRA. Carotenoids are also widely used in cosmetics preparations due to their skin moisturizing properties [361]. Carotenoids also have well-known UV light protective effects in human beings, functioning as a screen against solar radiation [362,363]. This activity is directly linked to their antioxidative properties and has been the focus of several studies. Indeed, diets rich in carotenoids, particularly in β-carotene, have been related to some resistance when exposed to sunlight [364,365].

The intake of supplements containing retinoids should be consulted with experts before use. Importantly, some studies have indicated the potential utility of retinoids as therapeutic agents against Alzheimer’s disease [247,366].

## 8. Hypervitaminosis and Vitamin A Toxicity

Vitamin A toxicity is a rather rare condition, but it can happen due to an enhanced intake of vitamin A or even after retinoid administration for therapeutic purposes. As specified previously, retinoid toxicity can appear both after oral and/or topical exposure [367,368].

Hypervitaminosis is considered when the blood concentration of retinol in the plasma is higher than 2.09 µM. Toxicity is commonly related to the misuse of dietary supplements but can also appear after an increased intake of food rich in preformed vitamin A (liver, eggs, etc.) [11,325]. Chronic toxicity can appear after a long-term intake of 10 mg/day of vitamin A for several months in adults and 7.5–15 mg/day in children. In general, toxicity is uncommon in intakes lower than 30 mg/day (25,000–30,000 IU/day) [11]. Acute vitamin A toxicity usually appears after more than 500 mg/day in adults, 100 mg/day in children or 30 mg/day in infants. However, these cutoff levels are decreased in individuals with heavy alcohol consumption or with kidney failure [90,112]. In addition, mild adverse effects have been observed in vitamin A supplementation (loose stools, headache, irritability, fever, nausea and vomiting), they are rare and typically resolved quickly by discontinuation of vitamin A intake.

Hypervitaminosis A may be manifested by several symptoms, which also depend on age and hepatic function. A sudden excessive consumption of vitamin A leads to acute poisoning. The main symptoms observed in acute toxicity are nausea, irritability, reduced appetite, vomiting, blurry vision, headaches, hair loss, muscle pain, papilledema, hemorrhage, weakness, drowsiness and altered mental status [369,370]. These symptoms are quite frequent, whereas intracranial hypertension rarely appears. Idiopathic intracranial hypertension (pseudotumor cerebri), a syndrome characterized by headache, blurred vision, confusion and increased intracerebral pressure, is also being reported in patients with excessive consumption of vitamin A or by those treated with isotretinoin [357]. Hypertriglyceridemia is the most common biochemical adverse effect detected after retinoid administration. This appears several weeks after the initiation of treatment. Eventually, these elevated triglyceride levels lead to liver damage, which, as a consequence, causes fibrosis and hepatic stellate cell activation, leading to possible irreversible liver damage. On the other hand, oral retinoids can cause cracked lips, headache, flushing, stomach pain, dizziness and loss of coordination [371,372].

Sebum production is decreased as a consequence, which reduces epidermal thickness and alters the barrier function of the skin. These and other cutaneous effects (skin dryness, pruritus, overall and fingertip fissuring), including alopecia, also appear in this type of toxicity but usually disappear upon cessation of the treatment. In chronic patients, in addition to the acute symptoms described above, insomnia, hypothyroidism, bone destruction, anemia, fatigue, diarrhea, dry and pruritic skin, skin and mucosa desquamation, hepatosplenomegaly, liver hypertrophy, hypertension, fibrosis, sclerosis and cirrhosis can appear [138,267].

In addition, topical administration of retinoids can be associated with significant symptoms. Topical retinoids can cause skin redness (erythema), skin peeling (secondary to the hyper-proliferation of the epidermis) and discomfort. Other adverse effects include transient hypopigmentation or hyperpigmentation, psoriasis, allergic contact dermatitis and ectropion (the eyelids turned outwards) [245,357].

Importantly, retinoid overdose can cause the so-called “retinoic acid syndrome”. This condition manifests as acute respiratory distress with dyspnea, pleural and pericardial effusions, fever, weight gain, edema, and even multiorgan failure [373].

Generally, treating hypervitaminosis is based on the discontinuation of vitamin intake. Most of the signs and symptoms (acute retinoid toxicity, hypertriglyceridemia, skin and CNS symptoms) disappear gradually after intake discontinuation. Nevertheless, some of these symptoms, such as skin desquamation, remain evident for several months. Acetazolamide administration alleviates intracranial pressure. However, irreversible CNS consequences may occur [371,374]. Ophthalmic dryness following hypervitaminosis is managed with eye drops.

Retinoids are also teratogenic. Some of the teratogenic effects of retinoid administration in pregnant women include craniofacial, cardiac, thymic and CNS abnormalities in the fetus [375]. Since oral retinoids of both animal and synthetic origin are teratogenic, women treated with synthetic retinoids should avoid getting pregnant for a considerable period even after treatment discontinuation. In some cases, e.g., after isotretinoin treatment, 2 years is recommended [119]. However, their use in pregnant women can be indicated in cases where the benefit provided by the therapy is greater than the risk and always under medical supervision. In children (<5 years) at risk of deficiency, vitamin A supplementation is recommended and has proved to decrease mortality in this patient group. Retinol levels should be monitored in patients suffering from hepatic and renal diseases, alcoholism, and acne. In patients treated with vitamin A supplements, triglyceride levels should be periodically controlled. Liver enzyme elevations are usually mild and reversible, but should also be monitored. Bone quality monitoring is only recommended in patients receiving multiple courses of isotretinoin or if the patient is undergoing a long-term treatment. If the patient has a history of kidney disease, renal function should be monitored during treatment.

Carotenoids are considered safe compounds. Significant toxicity is very rare. In cases of excessive carotenoid intake, orange/yellowish skin coloration appears (carotenoderma or carotenemia). These symptoms are fully reversible; they disappear with time after the discontinuation of carotenoid intake. Diagnostically, this condition is differentiated from jaundice due to a lack of scleral pigmentation. The likely reason is the above mentioned regulation of the metabolism of provitamins A. However, carotenemia has been rarely related to the appearance of nephrotic syndrome, liver diseases, hypothyroidism and other conditions [376]. On the other hand, controversial data were published by a Finnish study reporting enhanced cardiovascular mortality and even pulmonary cancer incidence in a cohort of smoking individuals with a high β-carotene intake [197]. The relationship between β-carotene and lung cancer was also described in Section 4.2.1.

## 9. Conclusions

A significant percentage of the population regularly consumes dietary supplements and preparations containing multiple micronutrients, including vitamins. It is a common belief that these preparations are safe products, but several thousand intoxications are reported every year due to using these products. Vitamin A is a good example. On one hand, it is necessary for correct body functioning, but on the other, its excessive intake is manifested by several symptoms of toxicity.

The popularization of Internet use and easy access to tons of information poses a risk to widespread misinformation. The main outcome is an enhanced risk of misusing dietary supplements by those seeking a healthier lifestyle. Among these groups, pregnant women, children and seniors should preferentially consult professionals before taking any of these supplements, which could have deleterious effects on their health. A typical example is β-carotene, whose intake is mostly considered to be rather positive. However, its use in dietary supplements should be avoided by smokers. On the other hand, the administration of different drugs with vitamin A properties is useful to treat several diseases, but medical specialists should always guide such indications.

Given the importance of vitamin A in multiple crucial physiological processes, its deficiency can pose a serious health challenge, even leading to death in the most serious cases. At the same time, it can lead to serious health issues in high-dose situations. This review summarized the body of knowledge surrounding vitamin Ain a complex way. In addition to well-established knowledge, mainly regarding the origin and toxic outcomes of the micronutrient, this manuscript described in detail the molecular pathways for its functioning in the human body and the latest methods used to assess its amounts in human samples.

## Figures and Tables

**Table 1:** Content of β-carotene in selected sources.

Source	Latin Name	Family	β-Carotene Content	Refs.
Orange-red vegetables				
Sweet potato	*Ipomoea batatas* (L.) Lam.	Convolvulaceae	20–22,600 ^a^	[12,18,22,35]
Bitter gourd	*Momordica charantia* L.	Cucurbitaceae	17,040 ^b^	[36]
Winter squash (butternut)	*Cucurbita moschata* Duchesne	Cucurbitaceae	12,340–15,770 ^a^	[21]
Chili pepper	*Capsicum annuum* L., *C. chinense* Jacq., *C. frutescens* L.	Solanaceae	100–15,400 ^a^	[12,37]
Carrot	*Daucus carota* L.	Apiaceae	4350–8840 ^a^	[35,38]
Pumpkins	*Cucurbita maxima* Duchesne	Cucurbitaceae	70–6070 ^a^	[12,17,35,38]
Cantaloupe	*Cucumis melo* L.	Cucurbitaceae	2448–3861 ^a^	[39]
Red pepper	*Capsicum annuum* L.	Solanaceae	1441–2390 ^a^	[12]
Tomato	*Solanum lycopersicum* L.	Solanaceae	59–1500 ^a^	[12,35,38]
Green vegetables				
Drumstick leaves	*Moringa oleifera* Lam.	Moringaceae	19,700 ^a^	[35]
Amaranth	*Amaranthus gangeticus* L.	Amaranthaceae	8600 ^a^	[35]
Kale	*Brassica oleracea* var. *acephala* DC.	Brassicaceae	1020–10,000 ^a^	[12,40]
Garden rocket	*Eruca vesicaria* (L.) Cav.	Brassicaceae	7960 ^a^	[41]
Chicory	*Cichorium intybus* L.	Asteraceae	3940–7310 ^a^	[41]
Wild rocket	*Diplotaxis tenuifolia* (L.) DC.	Brassicaceae	7010 ^a^	[41]
Dandelion	*Taraxacum officinale* (L.) Weber ex F.H. Wigg.	Asteraceae	6340 ^a^	[41]
Onion leaf	*Allium cepa* L.	Amaryllidaceae	4900 ^a^	[35]
Coriander	*Coriandrum sativum* L.	Apiaceae	4800 ^a^	[35]
Parsley	*Petroselinum crispum* (Mill.) Fuss	Apiaceae	4440–4680 ^a^	[12]
Spinach	*Spinacia oleracea* L.	Amaranthaceae	3100–4810 ^a^	[12]
Endive	*Cichorium endivia* L.	Asteraceae	1340–4350 ^a^	[12]
Cress	*Lepidium sativum* L.	Brassicaceae	2720–3690 ^a^	[12]
Leek	*Allium ampeloprasum* L.	Amaryllidaceae	3190 ^a^	[12]
Lettuce	*Lactuca sativa* L.	Asteraceae	870–2960 ^a^	[12]
Broccoli	*Brassica oleraceae* var. *italica* Plenck.	Brassicaceae	291–1750 ^a^	[12]
Fruits				
Apricot	*Prunus* sect. *Armeniaca* (Scop.) Koch	Rosaceae	585–3800 ^a^	[12,19]
Mango	*Mangifera indica* L.	Anacardiaceae	109–3210 ^a^	[12,35,42]
Persimmon	*Diospyros kaki* L.f.	Ebenaceae	3000 ^b^	[36]
Dates	*Phoenix* sp. L.	Arecaceae	2950 ^a^	[35]
Guava	*Psidium guajava* L.	Myrtaceae	1–2669 ^a^	[12,35]
Red grapefruit	*Citrus paradisi* Macfad.	Rutaceae	2580 ^a^	[27]
Papaya	*Carica papaya* L.	Caricaceae	190–1050 ^a^	[35,42,43]
Cereals				
Golden rice	*Oryza sativa* L.	Poaceae	160–3700 ^a^	[44]
Maize	*Zea mays* L.	Poaceae	171–1500 ^a^	[38,44]
Medicinal plants and herbs				
Rose hips	*Rosa rubiginosa* L.	Rosaceae	3600 ^a^	[45]
Marigold flowers	*Calendula officinalis* L.	Asteraceae	940–20,600 ^a^	[46]
Dill	*Anethum graveolens* L.	Apiaceae	5450 ^a^	[12]
Basil	*Ocimum basilicum* L.	Lamiaceae	4820 ^a^	[12]
Others				
Spirulina	*Spirulina* sp. Turpin ex Gomont	Spirulinaceae	184,100–272,500 ^a^	[31]
Sea buckthorn oil	*Elaeagnus rhamnoides* (L.) A. Nelson	Elaeagnaceae	16,740 ^c^	[47]
Red palm oil	*Elaeis guineensis* Jacq.	Arecaceae	5000–5602 ^c^	[48]

^a^ µg/100 g of fresh weight, ^b^ CE-β-carotene equivalent (µg/100 g of fresh weight)—also includes other carotenoids (content of β-carotene + 1/2 content of other vitamin A active carotenoids), ^c^ µg/100 g of oil.

**Table 2:** Summary of methods for determination of retinoids and carotenoids in human biological materials.

Technique	Sensitivity (nmol/L)	Matrix	Analytes	Advantages	Disadvantages	References
HPLC-UV-vis/DAD	^1^ 0.1 × 10^−3^–209.46	Serum, plasma, seminal plasma, mouse embryos and kidney, dried whole blood spots, breast milk, red blood cells, adipose tissue	Retinoids (retinol, retinal, RA, retinyl esters) and carotenoids	Usually small sample volume (30–200 µL) Combination with automation and online sample preparation Short analysis times in some multicomponent analyses Some methods have comparable sensitivity to MS detection	Long analysis time with a complicated gradient Use of a large volume of toxic solvents as the mobile phase and in sample preparation procedures, mainly in carotenoids analyses	[279,280,281,282,283,284,285,286,287,288,289,290,291,292,293,294,295]
HPLC-FLD	^1^ 2.3–34.91	Plasma, breast milk	Retinol		Not often used No sufficient data	[296,297]
LC–MS LC–MS/MS	^2^ 2 × 10^−6^–261.83	Urine, plasma, amniotic fluid, tears, serum	Retinol, RA	Usually, simple sample preparation procedures	In the case of retinoic acid usage of large volumes of toxic solvents in sample preparation procedures with poor recovery	[298,299,300,301,302,303]
SFC-MS/MS	^1^ 0.09 × 10^−6^–70.31	Whole blood, plasma, serum, colostrum	Carotenoids, apocarotenoids, epoxycarotenoids	Small sample volume (10–200 µL) Short analysis times in multicomponent analyses Combination with online SFE or robotic SLE		[304,305,306,307]
HPLC-ECD	^1^ 0.4 × 10^−3^–314.19	Serum, rat plasma, cervical tissue	Retinol, RA, carotenoids	Small sample volume (20–200 µL), Short analysis times in multicomponent analyses of retinol and RA	Long analysis time of carotenoids Toxic solvents usage	[308,309,310]
ELISA kits	^1^ 0.11–279.38	Whole blood, serum, cell lysates, plasma, tissues, other human liquids, colostrum	Retinol, β-carotene	One kit for various matrices (serum, plasma, other biofluids, cell lysates)	For research only Cross-reactivity with retinol analogs Time and money consuming for small sample series	[311,312,313,314,315,316,317,318,319]
HPLC/UHPLC-UV kits	^2^ 23.62–1174.5	Plasma, serum	Retinol, carotenoids	Small sample volume (50–250 µL) Some kits: possible to combine with a 96-well plate format 96 samples in 30 min Some kits: available in UHPLC mode–3 analytes in 3.5 min	Some kits have long analysis times and use large sample volumes (400 µL) Only for serum or plasma	[320,321,322,323,324]

^1^ LOD—limit of detection, ^2^ LLOQ—lower limit of quantification. DAD—diode array detection; ECD—electrochemical detection; ELISA enzyme-linked immunosorbent assay; FLD—fluorescence detection; HPLC—high-performance liquid chromatography; LC–MS—coupling of liquid chromatography and mass spectrometry; RA—retinoic acid; SFC-MS—coupling of supercritical fluid chromatography and mass spectrometry; SFE—supercritical fluid extraction; SLE—solid-supported liquid–liquid extraction; UHPLC—ultra-high-performance liquid chromatography; UV-vis—ultraviolet/visible detection.

## Supplementary Materials

The following are available online at https://www.mdpi.com/article/10.3390/nu13051703/s1, Table S1: A detailed summary of the methods used to determine retinoids and carotenoids in human biological materials.
