# Supplementary material for: Vitamin A Update: Forms, Sources, Kinetics, Detection, Function, Deficiency, Therapeutic Use and Toxicity
Source: Nutrients. 2021 May 18;13(5):1703. doi: 10.3390/nu13051703 (PMC8157347; doi:10.3390/nu13051703)
Supplement: Supplementary file 1 [file nutrients-13-01703-s001.zip › Vitamin A - Supplementary Material.pdf]

# Supplementary materials - Carazo et al. 2021, Nutrients

**Table S1.** Detailed summary of methods for determination of retinoids and carotenoids in human biological materials

| Technique | Sensitivity (nmol/L) | Matrix | Analytes | Advantages | Disadvantages | Reference |
|---|---|---|---|---|---|---|
| HPLC-UV/VIS/PDA | [1]5.43 – 49.93 | serum | 9 carotenoids | * combination with SPE<br>* 9 analytes in 17 min | * usage of toxic solvents (THF) | [1] |
| | [2]20 – 120 | plasma | retinol, carotenoids, 10 analytes | * single LLE<br>* usage only 200 μL of sample | * complicated gradient elution in 32 min | [2] |
| | [1]0.2 – 2 | serum, seminal plasma | retinol, ATRA, carotenoids | * very simple sample preparation<br>* various body fluids<br>* only 250 μL<br>* 15 analytes in 30 min | * complicated gradient elution | [3] |
| | [2]4.52 – 8.73 | plasma | retinol, retinyl esters (palmitate, stearate, oleate, linoleate) | * automated column switching usage | * 5 analytes in 25 min<br>* complicated mobile phase | [4] |
| | [2]2.29 – 4.19 | mouse embryos and kidney, rat plasma | retinol, retinoic acids, retinyl palmitate, 6 analytes | * automated SPE – HPLC | * 6 retinoids in 42 min<br>* complicated multilinear gradient | [5] |
| | [2]1.75 | serum | retinol | * minimized human intervention<br>* fully automated<br>* 35 min per sample | * 2 mL of human serum<br>* triple SPE, double LLE<br>* 5 analytes in 15 min | [6] |
| | [1]3.49 | serum | retinol | | * complicated sample preparation using large volumes of solvents | [7] |
| | [2]87.28 | serum | retinol | * 100 μL sample | * 3 analytes in 7 min | [8] |
| | [1]100 | dried whole blood spots | retinol | * 2 analytes in 5 min<br>* DBS – 30 μL of sample | * toxic mobile phase | [9] |
| | [1]4 | breast milk | retinol | * 2 analytes in 1.8 min | | [10] |

| | | | | | | |
|---|---|---|---|---|---|---|
| | [1]0.1 × $10^{-3}$ – 0.25 × $10^{-3}$ | red blood cells | carotenoids | * same sensitivity with DAD as APCI-MS<br>* both used in this study | * 8 analytes in 30 min<br>* toxic mobile phase<br>* large volumes of toxic solvents in sample preparation procedure | [11] |
| | [2]4 – 2.8 | serum | retinol, carotenoids | * 15 analytes in 13 min by isocratic elution<br>* 200 μL of sample<br>* sensitivity | * large volumes of toxic solvents in sample preparation procedure | [12] |
| | [2]11.01 – 54.01 | serum | carotenoids | * 30 analytes in 45 min | * toxic mobile phase<br>* large volumes of sample and toxic solvents in sample preparation procedure | [13] |
| | [2]69.82 | plasma | retinol | * 100 μL of sample<br>* 4 analytes in 6 min | | [14] |
| | [3]104.73 | plasma | retinol | * 3 analytes in 4 min<br>* 200 μL of sample | | [15] |
| | [1]1.5 | seminal plasma | retinol | * 200 μL of sample | * 4 analytes in 13 min<br>* large volumes of toxic solvents in sample preparation procedure | [16] |
| | [2]209.46<br>[2]33.51 – 37.25 | adipose tissue, plasma | retinol carotenoids | * toxic mobile phase, but in comparison with other methods low consumption per analysis<br>* 250 μL of sample | | [17] |
| HPLC-FLD | [1]34.91 | plasma | retinol, 2 analytes | * miniaturized SPE in syringe using nanofibers<br>* 300 μL sample | * 2 analytes in 17 min | [18] |
| | [1]2.3 | breast milk, serum | retinol, 2 analytes | * 2 analytes in 1.4 min | | [19] |
| LC-MS | [1]10 | urine | retinol, 2 analytes | * 2 analytes in 4 min<br>* simple sample preparation<br>* MRM | * low retinol recovery | [20] |
| | [1]7.51 | plasma | retinol, 3 analytes | * miniaturized SPE, DBS<br>* 100 μL of sample<br>* MRM | * 4 analytes in 10 min | [21] |
| | [3]261.83 | plasma amniotic fluid | retinol | * simple sample preparation | * 5 analytes in 15 min | [22] |

| | | | | | | |
|---|---|---|---|---|---|---|
| | [1]195.49 | tears, serum | retinol,<br>7 analytes | * tears and blood | | [23] |
| | [3]76.80 | serum | retinol,<br>4 analytes | * Unisprey as ionisation source less prone to ME - sensitivity without complex sample preparation<br>* MRM | | [24] |
| | [3]2 – 40 × 10-6 | serum | retinoic acids | * high sensitivity | * 5 analytes in 14.5 min<br>* large volumes of toxic solvents in sample preparation procedure with poor recovery of hydroxylated metabolites of RA | [25] |
| SFC-MS | [2]14.90 – 70.31 | whole blood | 15 carotenoids and apocarotenoids | * 15 analytes in 20 min<br>* online SFE<br>* only 10 μL of sample | * 4 carotenoids quantified | [26] |
| | [2]0.36 – 7.03 | plasma | carotenoids | * 14 analytes in 10 min<br>* robotic SLE<br>* 200 μL of sample<br>* green mobile phase for carotenoids separation | | [27] |
| | [1]0.09 –<br>0.19 × 10-6 | serum | carotenoids and epoxycarotenoids | * 13 analytes in 15 min<br>* 100 μL of sample | * expensive standards of epoxycarotenoids<br>* toxic solvents used in sample preparation<br>* epoxycarotenoids not quantified | [28] |
| | [2]27.4 – 54.3 | colostrum | 7 carotenoids and 25 apocarotenoids | * 32 analytes in 30 min<br>* 20 μL of sample | * apocarotenoids not quantified | [29] |
| HPLC-ECD | [2]0.67 – 2.81 | serum | retinol,<br>ATRA, 13-cis-RA<br>retinyl acetate, retinal | * 6 analytes in 12 min<br>* sensitivity<br>* 200 μL of sample | | [30] |
| | [1]314.19 | rat plasma | retinol | * 7 analytes in 14 min | * double LLE | [31] |
| | [1]0.4 × 10-3 | serum, cervical tissue | carotenoids | * sensitivity<br>* 20 μL sample | * 5 analytes in 30 min but corresponds to the date of publication<br>* toxic solvents | [32] |

| | | | | | | |
|---|---|---|---|---|---|---|
| FIA-CL | [1]80.29 | serum | retinol | * suitable only for pharmaceuticals | * interferences in biomatrix – necessary to use HPLC – impractical<br>* fivefold LLE | [33] |
| CE-MEKC-UV | [2]2618.26 | serum | retinol | | * 3 analytes in 20 min<br>* 1 mL of sample<br>* toxic solutions used in sample preparation<br>* 5 min flushing of the capillary between each run | [34] |
| Affinity CE | [1]0.02 | serum albumins solution | retinol,<br>RA for binding study with serum albumins | * high sensitivity cell usage | * 8 min flushing of the capillary between each run<br>* not real samples used | [35] |
| Biosensors | [1]15.3 | synthetic plasmatic serum | 13-cis-RA | | * applied only at artificial serum | [36] |
| ELISA kits | [3]100 | serum, cell lysates, plasma, tissues | retinol | * serum, cell lysates, plasma, tissues<br>* range 100 – 10 000 nmol/L | * necessary to predict concentrations and dilute<br>* for research only<br>* cross reactivity with retinol analogues<br>* time and money consuming for small sample series | [37] |
| | <[1]173.50 | serum, plasma, human liquids | retinol | * serum, plasma, human liquids<br>* range 431 – 34 910 nmol/L | * for research only<br>* cross reactivity with retinol analogues<br>* time and money consuming for small sample series | [38] |
| | [1]0.182 | serum, cell lysates, plasma, tissues | retinol | * serum, cell lysates, plasma, tissues | * for research only<br>* cross reactivity with retinol analogues<br>* time and money consuming for small sample series | [39] |
| | [1]0.105 | serum, plasma, biofluids, cell lysates | retinol | * serum, plasma, biofluids, cell lysates<br>* sensitivity | * for research only<br>* cross reactivity with retinol analogue<br>* time and money consuming for small sample series | [40] |

| | | | | | | |
|---|---|---|---|---|---|---|
| | [1]1.63 | serum, plasma, biofluids, cell lysates | retinol | * serum, plasma, biofluids, cell lysates | * for research only<br>* cross reactivity with retinol analogues<br>* time and money consuming for small sample series | [41] |
| | [1]1.63 | serum, plasma | retinol | | | [42] |
| | [1]10 | serum, plasma, biofluids, cell lysates | β-carotene | * serum, plasma, biofluids, cell lysates<br>* 40 μL<br>* range 50 – 10 000 nmol/L | * for research only<br>* cross reactivity with carotenoids analogues<br>* time and money consuming for small sample series | [43] |
| | [3]279.38 | colostrum, cattle whole blood and serum | β-carotene | * portable On-site quantitative testing<br>* range 279 – 27 938 nmol/L | * total carotenoids<br>* only in animal BM<br>* 400 μL BM<br>* for research only | [44] |
| HPLC-UV kit | [2]69.8 | plasma, serum | retinol | * simple sample preparation | * 3 analytes in 10 min<br>* expensive for small sample series | [45] |
| | [3]23.62 | plasma, serum | carotenoids | * 7 analytes in 10 min<br>* simple sample preparation<br>* 100 μL of sample | * expensive for small sample series | [46] |
| | [1]174.5 | plasma, serum | retinol | * simple sample preparation<br>* 250 μL of sample | * 3 analytes in 10 min<br>* expensive for small sample series | [47] |
| HPLC/UHPLC-UV kit | [2]69.8 | plasma, serum | retinol | * possible to combine with 96well plate format<br>* 96 samples in 30 min<br>* 50 μL of sample<br>* available in UHPLC mode: 3 analytes in 3.5 min | * 3 analytes in 9 min in HPLC mode<br>* expensive for small sample series | [48] |
| UHPLC-UV kit | [2]139.6 | plasma, serum | retinol | * 50 μL of sample | * 3 analytes in 1 min | [49] |
| Raman spectroscopy | not specified | skin, plasma | carotenoids | * no sample preparation<br>* noninvasive skin measurement<br>* no toxic waste<br>* correlation with levels in blood measured by HPLC | * autofluorescence (sample dependent)<br>* sensitivity not specified | [50-52] |

1 LOD Limit of Detection, 2 LOQ Limit of Quantification, 3 LLOQ Lower Limit of Quantification

APCI Atmospheric Pressure Chemical Ionization; ATRA All-trans Retinoic Acid; BM Biological Material; CE Capillary Electrophoresis; DAD Diode Array Detection; DBS Dry Blood Spot; ECD Electrochemical Detection; ELISA Enzyme-Linked ImmunoSorbent Assay; FIA-CL Flow-Injection Analysis with Chemiluminescence detection; FLD Fluorescence Detection; HPLC High Performance Liquid Chromatography; LC-MS Coupling of Liquid Chromatography and Mass Spectrometry; LLE Liquid-Liquid Extraction; ME Matrix Effects; MEKC Micellar Electrokinetic Chromatography; MRM Multiple Reaction Monitoring; MS Mass Spectrometer; PDA PhotoDiode Array Detection; RA Retinoic Acid; SFC-MS Coupling of Supercritical Fluid Chromatography and Mass Spectrometry; SFE Supercritical Fluid Extraction; SLE Solid Supported Liquid-Liquid Extraction; SPE Solid Phase Extraction; THF Tetrahydrofuran; UHPLC Ultra-High Performance Liquid Chromatography; UV/VIS Ultraviolet/Visible Detection;

1. Ferreiro-Vera, C.; Mata-Granados, J.M.; Quesada Gomez, J.M.; Luque de Castro, M.D. On-line coupling of automatic solid-phase extraction and HPLC for determination of carotenoids in serum. *Talanta* **2011**, *85*, 1842-1847, doi:10.1016/j.talanta.2011.07.031.
2. Boulet, L.; Alex, B.; Clavey, N.; Martinez, J.; Ducros, V. Simultaneous analysis of retinol, six carotenoids, two tocopherols, and coenzyme Q10 from human plasma by HPLC. *J Chromatogr B Analyt Technol Biomed Life Sci* **2020**, *1151*, 122158, doi:10.1016/j.jchromb.2020.122158.
3. Lazzarino, G.; Longo, S.; Amorini, A.M.; Di Pietro, V.; D'Urso, S.; Lazzarino, G.; Belli, A.; Tavazzi, B. Single-step preparation of selected biological fluids for the high performance liquid chromatographic analysis of fat-soluble vitamins and antioxidants. *J Chromatogr A* **2017**, *1527*, 43-52, doi:10.1016/j.chroma.2017.10.053.
4. Hartmann, S.; Froescheis, O.; Ringenbach, F.; Wyss, R.; Bucheli, F.; Bischof, S.; Bausch, J.; Wiegand, U.W. Determination of retinol and retinyl esters in human plasma by high-performance liquid chromatography with automated column switching and ultraviolet detection. *J Chromatogr B Biomed Sci Appl* **2001**, *751*, 265-275, doi:10.1016/s0378-4347(00)00481-3.
5. Ruhl, R.; Schweigert, F.J. Automated solid-phase extraction and liquid chromatographic method for retinoid determination in biological samples. *J Chromatogr B Analyt Technol Biomed Life Sci* **2003**, *798*, 309-316, doi:10.1016/j.jchromb.2003.10.001.
6. Mata-Granados, J.M.; Luque De Castro, M.D.; Quesada, J.M. Fully automated method for the determination of 24,25(OH)2 and 25(OH) D3 hydroxyvitamins, and vitamins A and E in human serum by HPLC. *J Pharm Biomed Anal* **2004**, *35*, 575-582, doi:10.1016/j.jpba.2004.01.027.
7. Xuan, R.; Wang, T.; Hou, C.; Li, X.; Li, Y.; Chen, Y.; Gao, Y.; Qiu, D.; Xiao, X.; Zhang, L., et al. Determination of vitamin A in blood serum based on solid-phase extraction using cetyltrimethyl ammonium bromide-modified attapulgite. *J Sep Sci* **2019**, *42*, 3521-3527, doi:10.1002/jssc.201900778.
8. Pan, Q.; Shen, M.; Yu, T.; Yang, X.; Li, Q.; Zhao, B.; Zou, J.; Zhang, M. Liquid chromatography as candidate reference method for the determination of vitamins A and E in human serum. *J Clin Lab Anal* **2020**, 10.1002/jcla.23528, e23528, doi:10.1002/jcla.23528.
9. Erhardt, J.G.; Craft, N.E.; Heinrich, F.; Biesalski, H.K. Rapid and simple measurement of retinol in human dried whole blood spots. *J Nutr* **2002**, *132*, 318-321, doi:10.1093/jn/132.2.318.
10. Plisek, J.; Kasparova, M.; Solichova, D.; Krcmova, L.; Kucerova, B.; Sobotka, L.; Solich, P. Application of core-shell technology for determination of retinol and alpha-tocopherol in breast milk. *Talanta* **2013**, *107*, 382-388, doi:10.1016/j.talanta.2013.01.031.
11. Nakagawa, K.; Kiko, T.; Hatade, K.; Asai, A.; Kimura, F.; Sookwong, P.; Tsuduki, T.; Arai, H.; Miyazawa, T. Development of a high-performance liquid chromatography-based assay for carotenoids in human red blood cells: application to clinical studies. *Anal Biochem* **2008**, *381*, 129-134, doi:10.1016/j.ab.2008.06.038.
12. Thibeault, D.; Su, H.; MacNamara, E.; Schipper, H.M. Isocratic rapid liquid chromatographic method for simultaneous determination of carotenoids, retinol, and tocopherols in human serum. *J Chromatogr B Analyt Technol Biomed Life Sci* **2009**, *877*, 1077-1083, doi:10.1016/j.jchromb.2009.02.051.
13. Hsu, B.Y.; Pu, Y.S.; Inbaraj, B.S.; Chen, B.H. An improved high performance liquid chromatography-diode array detection-mass spectrometry method for determination of carotenoids and their precursors phytoene and phytofluene in human serum. *J Chromatogr B Analyt Technol Biomed Life Sci* **2012**, *899*, 36-45, doi:10.1016/j.jchromb.2012.04.034.
14. Bell, E.C.; John, M.; Hughes, R.J.; Pham, T. Ultra-performance liquid chromatographic determination of tocopherols and retinol in human plasma. *J Chromatogr Sci* **2014**, *52*, 1065-1070, doi:10.1093/chromsci/bmt161.
15. Yuan, C.; Burgyan, M.; Bunch, D.R.; Reineks, E.; Jackson, R.; Steinle, R.; Wang, S. Fast, simple, and sensitive high-performance liquid chromatography method for measuring vitamins A and E in human blood plasma. *J Sep Sci* **2014**, *37*, 2293-2299, doi:10.1002/jssc.201301373.
16. Kandar, R.; Drabkova, P.; Myslikova, K.; Hampl, R. Determination of retinol and alpha-tocopherol in human seminal plasma using an HPLC with UV detection. *Andrologia* **2014**, *46*, 472-478, doi:10.1111/and.12103.
17. Gleize, B.; Steib, M.; Andre, M.; Reboul, E. Simple and fast HPLC method for simultaneous determination of retinol, tocopherols, coenzyme Q(10) and carotenoids in complex samples. *Food Chem* **2012**, *134*, 2560-2564, doi:10.1016/j.foodchem.2012.04.043.
18. Liu, Z.e.a. Solid phase extraction with electrospun nanofibers

for determination of retinol and $\alpha$-tocopherol in plasma. *Microchim Acta* **2009**, *2010*, 59-64, doi:10.1007/s00604-009-0263-y.

19. Kucerova, B.; Krcmova, L.; Solichova, D.; Plisek, J.; Solich, P. Comparison of a new high-resolution monolithic column with core-shell and fully porous columns for the analysis of retinol and alpha-tocopherol in human serum and breast milk by ultra-high-performance liquid chromatography. *J Sep Sci* **2013**, *36*, 2223-2230, doi:10.1002/jssc.201300242.
20. Kucerova, K.e.a. Determination of urinary retinol and creatinine as an early sensitive marker of renal dysfunction. *J Chromatogr A* **2019**, *1607*, doi:10.1016/j.chroma.2019.460390.
21. Zhang, H.e.a. Simultaneous determination of Vitamin A, 25-hydroxyl vitamin D 3 $\alpha$-tocopherol in small biological fluids by liquid chromatography-tandem mass spectrometry. *J Chromatogr B Analyt Technol Biomed Life Sci* **2018**, *15*, 1-8.
22. Le, J.; Yuan, T.F.; Zhang, Y.; Wang, S.T.; Li, Y. New LC-MS/MS method with single-step pretreatment analyzes fat-soluble vitamins in plasma and amniotic fluid. *J Lipid Res* **2018**, *59*, 1783-1790, doi:10.1194/jlr.D087569.
23. Khaksari, M.; Mazzoleni, L.R.; Ruan, C.; Kennedy, R.T.; Minerick, A.R. Data representing two separate LC-MS methods for detection and quantification of water-soluble and fat-soluble vitamins in tears and blood serum. *Data Brief* **2017**, *11*, 316-330, doi:10.1016/j.dib.2017.02.033.
24. Peersman, N.; Elslande, J.V.; Lepage, Y.; De Amicis, S.; Desmet, K.; Vermeersch, P. UPLC-MS/MS method for determination of retinol and alpha-tocopherol in serum using a simple sample pretreatment and UniSpray as ionization technique to reduce matrix effects. *Clin Chem Lab Med* **2020**, *58*, 769-779, doi:10.1515/cclm-2019-1237.
25. Arnold, S.L.; Amory, J.K.; Walsh, T.J.; Isoherranen, N. A sensitive and specific method for measurement of multiple retinoids in human serum with UHPLC-MS/MS. *J Lipid Res* **2012**, *53*, 587-598, doi:10.1194/jlr.D019745.
26. Zoccali, M.; Giuffrida, D.; Salafia, F.; Giofre, S.V.; Mondello, L. Carotenoids and apocarotenoids determination in intact human blood samples by online supercritical fluid extraction-supercritical fluid chromatography-tandem mass spectrometry. *Anal Chim Acta* **2018**, *1032*, 40-47, doi:10.1016/j.aca.2018.06.022.
27. Petruzziello, F.; Grand-Guillaume Perrenoud, A.; Thorimbert, A.; Fogwill, M.; Rezzi, S. Quantitative Profiling of Endogenous Fat-Soluble Vitamins and Carotenoids in Human Plasma Using an Improved UHPSFC-ESI-MS Interface. *Anal Chem* **2017**, *89*, 7615-7622, doi:10.1021/acs.analchem.7b01476.
28. Matsubara, A.; Uchikata, T.; Shinohara, M.; Nishiumi, S.; Yoshida, M.; Fukusaki, E.; Bamba, T. Highly sensitive and rapid profiling method for carotenoids and their epoxidized products using supercritical fluid chromatography coupled with electrospray ionization-triple quadrupole mass spectrometry. *J Biosci Bioeng* **2012**, *113*, 782-787, doi:10.1016/j.jbiosc.2012.01.017.
29. Zoccali, M.; Giuffrida, D.; Granese, R.; Salafia, F.; Dugo, P.; Mondello, L. Determination of free apocarotenoids and apocarotenoid esters in human colostrum. *Anal Bioanal Chem* **2020**, *412*, 1335-1342, doi:10.1007/s00216-019-02359-z.
30. Wang, L.H.e.a. Determination of retinoids in human serum, tocopherol and retinyl acetate in pharmaceuticals by RP-LC with electrochemical detection. *J Pharm Biomed Anal* **2001**, 785-793.
31. Hermans, N.; Cos, P.; Berghe, D.V.; Vlietinck, A.J.; de Bruyne, T. Method development and validation for monitoring in vivo oxidative stress: evaluation of lipid peroxidation and fat-soluble vitamin status by HPLC in rat plasma. *J Chromatogr B Analyt Technol Biomed Life Sci* **2005**, *822*, 33-39, doi:10.1016/j.jchromb.2005.05.040.
32. Ferruzzi, M.G.; Sander, L.C.; Rock, C.L.; Schwartz, S.J. Carotenoid determination in biological microsamples using liquid chromatography with a coulometric electrochemical array detector. *Anal Biochem* **1998**, *256*, 74-81, doi:10.1006/abio.1997.2484.
33. Asgher, M.; Yaqoob, M.; Waseem, A.; Nabi, A. Flow injection methods for the determination of retinol and alpha-tocopherol using lucigenin-enhanced chemiluminescence. *Luminescence* **2011**, *26*, 416-423, doi:10.1002/bio.1246.
34. Oledzka, I.; Kazmierska, K.; Plenis, A.; Kaminska, B.; Baczek, T. Capillary electromigration techniques as tools for assessing the status of vitamins A, C and E in patients with cystic fibrosis. *J Pharm Biomed Anal* **2015**, *102*, 45-53, doi:10.1016/j.jpba.2014.08.036.
35. El-Hady, D.A.; Albishri, H.M. Hyphenated affinity capillary electrophoresis with a high-sensitivity cell for the simultaneous binding study of retinol and retinoic acid in nanomolars with serum albumins. *J Chromatogr B Analyt Technol Biomed Life Sci* **2012**, *911*, 180-185, doi:10.1016/j.jchromb.2012.11.007.
36. Fernandes, V.Q.e.a. Determination of isotretinoin (13-cis-retinoic acid) using a sensor basedon reduced graphene oxide modified with copper nanoparticles. *J Elechem* **2020**, *856*, 1-8, doi:10.1016/j.jelechem.2019.113692.

37. My BioSource. Human Vitamin A Elisa kit. Availabe online: https://cdn.mybiosource.com/tds/protocol_manuals/000000-799999/MBS729269.pdf (accessed on 27 Jan 2021).
38. Abbexa. Vitamin A Elisa Kit. Availabe online: https://www.abbexa.com/vitamin-a-elisa-kit (accessed on 27 Jan 2021).
39. Aviva Systems Biology. Vitamin A Elisa kit. Availabe online: https://www.avivasysbio.com/pub/media/pdf/products/OKEH02573.pdf (accessed on 27 Jan 2021).
40. Kamiya Biomedical Company. Human Vitamin A Elisa K-Assay. Availabe online: https://www.kamiyabiomedical.com/pdf/KT-31968.pdf (accessed on 27 Jan 2021).
41. LifeSpan BioSciences. Human Vitamin A Elisa kit. Availabe online: https://www.lsbio.com/elisakits/manualpdf/ls-f10051.pdf (accessed on 27 Jan 2021).
42. Cusabio. Human Vitamin A Elisa kit. Availabe online: https://cdn.mybiosource.com/tds/protocol_manuals/000000-799999/MBS729269.pdf (accessed on 27 Jan 2021).
43. Laboratory, B.T. Human Beta-Caroten Elisa kit. Availabe online: http://www.bt-laboratory.com/product/human-beta-carotene-elisa-kit-2/ (accessed on 27 Jan 2021).
44. BioAnalyt. iCheck Karotene. Availabe online: https://www.bioanalyt.com/wp-content/uploads/2019/05/iCheck-Carotene_product-information_EN_2019.pdf (accessed on 27 Jan 2021).
45. Recipe. HPLC complete kit Vitamin AE in plasma/serum. Availabe online: https://recipe.de/products/vitamin-a-e-serum/ (accessed on 27 Jan 2021).
46. Recipe. HPLC complete kit Beta-carotene in plasma/serum. Availabe online: https://recipe.de/products/%ce%b2-carotene-serum/ (accessed on 27 Jan 2021).
47. Immundiagnostic AG. Vitamin A/E HPLC kit. Availabe online: http://www.immundiagnostik.com/en/home/products/kits-assays/hplc-applications.html?tx_mokom01immunprodukte_pi1%5Ban%5D=KC1600&tx_mokom01immunprodukte_pi1%5Bag%5D=409&cHash=639cc97481 (accessed on 27 Jan 2021).
48. Chromsystems. Vitamin A and E in serum/plasma. Availabe online: https://chromsystems.com/en/vitamins-a-and-e-in-serum-plasma-hplc-34000.html (accessed on 27 Jan 2021).
49. Recipe. UHPLC Complete kit Vitamin A and E in serum/plasma. Availabe online: https://recipe.de/products/vitamin-a-e-serum-uhplc/ (accessed on 27 Jan 2021).
50. Zidichouski, J.A.; Mastaloudis, A.; Poole, S.J.; Reading, J.C.; Smidt, C.R. Clinical validation of a noninvasive, Raman spectroscopic method to assess carotenoid nutritional status in humans. *J Am Coll Nutr* **2009**, *28*, 687-693, doi:10.1080/07315724.2009.10719802.
51. Ermakov, I.V.; Ermakova, M.R.; Bernstein, P.S.; Chan, G.M.; Gellermann, W. Resonance Raman based skin carotenoid measurements in newborns and infants. *J Biophotonics* **2013**, *6*, 793-802, doi:10.1002/jbio.201200195.
52. Blume-Peytavi, U.; Rolland, A.; Darvin, M.E.; Constable, A.; Pineau, I.; Voit, C.; Zappel, K.; Schafer-Hesterberg, G.; Meinke, M.; Clavez, R.L., et al. Cutaneous lycopene and beta-carotene levels measured by resonance Raman spectroscopy: high reliability and sensitivity to oral lactolycopene deprivation and supplementation. *Eur J Pharm Biopharm* **2009**, *73*, 187-194, doi:10.1016/j.ejpb.2009.04.017.
